# Exerkine FNDC5/irisin‐enriched exosomes promote proliferation and inhibit ferroptosis of osteoblasts through interaction with Caveolin‐1

**DOI:** 10.1111/acel.14181

**Published:** 2024-04-30

**Authors:** Lin Tao, Jinpeng Wang, Ke Wang, Qichang Liu, Hongyang Li, Site Xu, Chunjian Gu, Yue Zhu

**Affiliations:** ^1^ Department of Orthopedics First Hospital of China Medical University Shenyang China

**Keywords:** Caveolin 1, exercise, exosome, ferroptosis, irisin, osteoporosis

## Abstract

Postmenopausal osteoporosis is a prevalent metabolic bone disorder characterized by a decrease in bone mineral density and deterioration of bone microstructure. Despite the high prevalence of this disease, no effective treatment for osteoporosis has been developed. Exercise has long been considered a potent anabolic factor that promotes bone mass via upregulation of myokines secreted by skeletal muscle, exerting long‐term osteoprotective effects and few side effects. Irisin was recently identified as a novel myokine that is significantly upregulated by exercise and could increase bone mass. However, the mechanisms underlying exercise‐induced muscle‐bone crosstalk remain unclear. Here, we identified that polyunsaturated fatty acids (arachidonic acid and docosahexaenoic acid) are increased in skeletal muscles following a 10‐week treadmill exercise programme, which then promotes the expression and release of FNDC5/irisin. In osteoblasts, irisin binds directly to Cav1, which recruits and interacts with AMP‐activated protein kinase α (AMPKα) to activate the AMPK pathway. Nrf2 is the downstream target of the AMPK pathway and increases the transcription of HMOX1 and Fpn. HMOX1 is involved in regulating the cell cycle and promotes the proliferation of osteoblasts. Moreover, upregulation of Fpn in osteoblasts enhanced iron removal, thereby suppressing ferroptosis in osteoblasts. Additionally, we confirmed that myotube‐derived exosomes are involved in the transportation of irisin and enter osteoblasts through caveolae‐mediated endocytosis. In conclusion, our findings highlight the crucial role of irisin, present in myotube‐derived exosomes, as a crucial regulator of exercise‐induced protective effects on bone, which provides novel insights into the mechanisms underlying exercise‐dependent treatment of osteoporosis.

## Graphical abstract

Exercise increases levels of arachidonic acid (AA) and DHA in skeletal muscle, promoting the expression and release of FNDC5/irisin via PGC1α. FNDC5/Irisin traffics to osteoblasts via exosomes and enters osteoblasts through caveolae‐mediated endocytosis dependent on Cav1. FNDC5/irisin then interacts with Cav1, which recruits AMPKα to activate the AMPK pathway. Nrf2 is thereby increased and promotes the transcription of HMOX1 and Fpn, resulting in increased proliferation and decreased ferroptosis of osteoblasts.

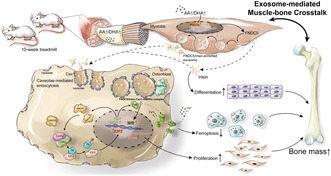

**Abbreviations**
AA: Arachidonic acid
ALP staining: Alkaline Phosphatase Staining
ALP: Alkaline phosphatase
AMPKα: Adenosine 5‘‐monophosphate (AMP)‐activated protein kinase α
ARS staining: Alizarin Red S Staining
BV/TV.Tb: Trabecular bone volume/tissue volume
Cav1: caveolin 1
CDK2: Cyclin dependent kinase 2
COL1: Collagen type I
CycA2: Cyclin‐A2
DHA: Docosahexaenoic acid
E2F2: Recombinant E2F Transcription Factor 2
FNDC5: Fibronectin Type III Domain Containing 5
Fpn: Ferroportin
GPX4: Glutathione Peroxidase 4
HMOX1: Heme oxygenase 1
IRI: Irisin
MB‐CM: Myotube‐conditioned medium
Nrf2: Nuclear factor erythroid 2‐related factor 2
OCN: Osteocalcin
OM: Osteogenic Medium
PA: Palmitic acid
PGC1α: Peroxisome proliferator‐activated receptor γ coactivator l alpha
RUNX2: Runt related transcription factor 2
Tb.BMD: Trabecular Bone Mineral Density
Tb.N: Trabecular number
Tb.Sp: Trabecular separation
Tb.Th: Trabecular Thickness
TEM: Transmission Electron Microscope

## 1 INTRODUCTION

Postmenopausal osteoporosis is a prevalent bone disease characterized by reduced bone mineral density (BMD) and structural deficiencies in bone tissues (Mullin et al., 2020). Osteoporotic fractures are one of the main causes of disability and mortality in elderly women. Currently, one commonly used treatment method in clinical practice is drug therapy, including antiresorptive medications (such as bisphosphonates and denosumab) and anabolic agents (such as teriparatide). However, current recommendations discourage the long‐term use of these antiosteoporotic medications, but discontinuation of these treatments could result in abrupt and rapid bone loss (Leder et al., 2009). Effective prevention and treatment of osteoporosis are crucial in reducing the risk of osteoporotic fractures. However, the currently available antiosteoporotic drugs, developed based on existing targets, have yielded unsatisfactory results, emphasizing the pressing necessity for the discovery of novel and more effective antiosteoporotic medications.

Exercise has long been acknowledged for its numerous long‐term health benefits (Fuller et al., 2012). Moreover, recent studies have highlighted the potential of various types of exercise to enhance bone mass in postmenopausal women, consequently reducing the risk of fractures (Ng et al., 2021). The potent anabolic effects of exercise in promoting bone mass exhibit various advantages, such as long‐term protective effects and the capability to regulate systemic metabolism with no side effects and lower costs compared with those of antiosteoporotic agents (Carson & Manolagas, 2015). However, continued exercise is necessary to maintain bone mass achieved through physical activity, which is notoriously difficult for postmenopausal women due to poor physical function and the high risk of falls (Hagman et al., 2018). Furthermore, bone injuries caused by long‐term exercise represent a challenge for postmenopausal women to maintain exercise. Hence, it is imperative to investigate the mechanisms involved in exercise and explore alternative approaches for the treatment of postmenopausal osteoporosis. Intertissue crosstalk is the major mechanism underlying the physiological effects of exercise (Garner et al., 2020). Skeletal muscles and bone are neighbouring tissues with tight connections. Notably, skeletal muscles function as major endocrine organs in response to exercise stimuli by secreting various hormones and cytokines, collectively referred to as myokines (Tezze et al., 2017). Myokines serve as important mediators of skeletal muscle‐bone to regulate bone mass (He et al., 2020). Irisin is a myokine that is processed from fibronectin type III domain containing 5 (FNDC5), a transmembrane precursor protein expressed in muscle, and then secreted into circulation (Reza et al., 2017). In recent studies, irisin expression was significantly increased after exercise and had protective effects on bone health, suggesting that irisin might have a crucial role in muscle‐bone crosstalk (Xue et al., 2022). However, the underlying mechanisms responsible for the stimulation of irisin secretion through exercise and the potential mechanisms by which irisin might contribute to the protective effects of bone mass are currently not definitively understood.

Exosomes are a subpopulation of extracellular vesicles (EVs) characterized by their sizes ranging from 30 to 150 nm that act as message transmitters in intercellular communication to transport lipids, messenger RNA, DNA, microRNA and proteins (Zhang et al., 2019). Furthermore, exosomes have been demonstrated to be involved in intertissue crosstalk in a tissue‐specific fashion (Hoshino et al., 2015). Exercise was recently proposed to be involved in intertissue crosstalk by promoting the secretion of exosomes, which could enable the contents of the vesicle to be stably maintained for long‐distance transport (Whitham et al., 2018). Circulating levels of exosomes increased in response to physical activity, followed by a reduction during the subsequent recovery phase (Frühbeis et al., 2015). Moreover, a previous study demonstrated that acute exercise could increase the biogenesis of multivesicular bodies and the release of exosomes in skeletal muscle (Garner et al., 2020). Irisin is typically believed to be cleaved at the cell membrane and then directly released into circulation (Boström et al., 2012). Recently, irisin was also shown to be present in circulating EVs secreted by skeletal muscles (Chi et al., 2022). Exercise could stimulate the release of irisin via EVs, which are then trafficked to blood vessels to delay vascular senescence. However, the role of irisin‐enriched exosomes derived from skeletal muscles in bone health remains unknown.

Here, we found that irisin‐enriched exosomes derived from skeletal muscle, which were increased via exercise and then trafficked to osteoblasts, could attenuate bone loss in ovariectomized mice by promoting proliferation and inhibiting ferroptosis of osteoblasts. Exosomes entered osteoblasts via caveolae‐mediated endocytosis, while interactions among FNDC5/irisin‐Cav1‐AMPKα were identified as the major mediators of the anabolic effects of irisin in promoting bone mass. In addition, we further confirmed that arachnoid acid and docosahexaenoic acid were increased in response to exercise and promoted the release of irisin in skeletal muscle. These results provide novel insights into the effects of exercise on bone health and offer a new perspective for the treatment of osteoporosis based on irisin‐enriched exosomes.

## 2 MATERIALS AND METHODS

### 2.1 Study design, patient recruitment and data collection

This study was approved by the Ethical Review Committee of First Hospital of China Medical University. Postmenopausal women aged 55–65 were recruited in this study from March to September 2021. Participants were further screened for eligibility and excluded if they had any of the following: (a) unable to participate in long‐term exercises, (b) lumbar spine or lower limb joint injury; (c) cognitive impairment; (d) uncontrolled cardiovascular disease; (e) malignant tumours; (f) receiving X‐ray or radiation therapy or (g) other medical history known to influence BMD. Prior to the study, all participants were provided with comprehensive information regarding the procedure and potential risks involved. Participants signed informed consent forms to indicate their understanding and agreement to participate.

### 2.2 Assessment of bone mineral density and serum levels of irisin

BMD for the lumbar spine and bilateral hips was measured by dual‐energy X‐ray absorptiometry. The average value of Chinese young women was used to calculate the T value. According to the WHO criteria, osteoporosis is diagnosed when BMD is 2.5 standard deviations below the average value of young individuals (*T*‐score < −2.5). On the other hand, osteopenia is identified when the *T*‐score falls between −2.5 and −1.0. Blood was taken via venipuncture at 7:00 am in the fasting state (≥10 h). After centrifugation, the serum was isolated and preserved at a temperature of −80°C until use. The serum concentration of irisin was measured using a Human Irisin ELISA kit (D711334‐0096) according to the manufacturer's instructions.

### 2.3 Animals

All 8‐week‐old female mice (C57BL6/J) were purchased from Beijing HFK Biotechnology Company (Beijing, China). After 1 week of arrival, mice were randomly divided into groups, anaesthetized and bilaterally ovariectomized (Ovx group) or sham‐operated (Sham group). In brief, the bilateral ovaries of the Ovx mice were surgically extracted by making a midline incision on the skin and flank incisions on the peritoneum. Mice in the sham operation group underwent a similar procedure but without the removal of the ovaries. All incisions were closed with stitches, and the mice were allowed to recover for 1 week. Ovx mice then underwent treadmill exercise or were treated with intraperitoneal injection of recombinant irisin according to the experiments.

#### 2.3.1 Treadmill

To investigate the impact of treadmill exercise on bone mass, mice were subjected to daily treadmill running sessions lasting 1 h at a speed of 10 m/min for 4 weeks (referred to as the treadmill group). At the end of the exercise regimen, all mice were euthanized, and the bilateral femurs, tibia, hindlimb muscles (including quadriceps, tibialis anterior, gastrocnemius, and soleus muscles), and serum were collected from each mouse. The serum was stored at −80°C, while the other tissues were rapidly frozen in liquid nitrogen and subsequently preserved at −80°C for further analysis.

#### 2.3.2 Irisin administration

To investigate the potential of irisin in mitigating bone loss in osteoporosis, Ovx mice were administered intraperitoneal injections of recombinant irisin at a dosage of 0.1 mg per kg body weight once a week for a duration of 4 weeks (referred to as the irisin group). Sham mice were intraperitoneally injected with the same amount of sterile water. On completion, all mice were sacrificed, and the bilateral femurs, tibia and serum of each mouse were collected. All samples were stored as mentioned above.

### 2.4 Micro‐CT

Microcomputed tomography (micro‐CT, SkyScan1276, Bruker, Germany) was performed in this study to evaluate the microstructure of the femur. Bone morphology parameters were also determined via micro‐CT, including BMD, bone volume fraction (bone volume/tissue volume, BV/TV), trabecular number (Tb.N), trabecular separation (Tb.Sp) and trabecular thickness (Tb.Th).

### 2.5 Untargeted metabolomics of bone tissues

To prepare the bone tissue samples, 100 mg of bone tissue was ground using liquid nitrogen and then resuspended in prechilled 80% methanol. After incubating on ice for 5 min, the samples were centrifuged at 15,000 ***g*** and 4°C for 20 min. The resulting supernatant was diluted to a final concentration containing 53% methanol and transferred to a fresh tube. This tube was then centrifuged again at 15,000 ***g*** and 4°C for 20 min. The supernatant obtained from the second centrifugation was injected into the LC‐MS/MS system for analysis.

The ultra performance liquid chromatography (UHPLC) ‐MS/MS analyses were conducted using a Vanquish UHPLC system (Thermo Fisher, Germany) coupled with an Orbitrap Q ExactiveTM HF mass spectrometer (Thermo Fisher, Germany) at Novogene Co., Ltd. (Beijing, China). The raw data files generated by UHPLC‐MS/MS were processed using Compound Discoverer 3.1 (CD3.1, Thermo Fisher) for peak alignment, peak picking and quantitation of each metabolite. Statistical analyses were performed using R (version R‐3.4.3), Python (version 2.7.6), and CentOS (CentOS release 6.6). Metabolites were annotated using the KEGG, HMDB and LIPID MAPS databases. Principal component analysis (PCA) and partial least squares discriminant analysis (PLS‐DA) were conducted using metaX. Univariate analysis (*t* test) was applied to determine the statistical significance (*p* value). Metabolites with VIP > 1, *p* < 0.05 and fold change ≥2 or ≤0.5 were considered as differentially abundant metabolites. Volcano plots were generated in R language to filter metabolites of interest based on fold change and *p* value.

### 2.6 Broad spectrum metabolomics of serum

To prepare the serum samples, 50 μL of serum was mixed with 300 μL of a 20% acetonitrile methanol internal standard extractant. The mixture was vortexed and then centrifuged at 12,000 rpm and 4°C for 10 min. The resulting supernatant was collected for analysis. The sample extracts were analysed using an LC‐electrospray ionisation (ESI)‐MS/MS system consisting of a UPLC (ExionLC AD) and a QTRAP® System (Sciex). Linear ion trap (LIT) and triple quadrupole (QQQ) scans were acquired on a QTRAP® LC‐MS/MS System, which was equipped with an ESI Turbo Ion Spray interface. The system operated in both positive and negative ion modes and was controlled by Analyst 1.6.3 software (Sciex). The ESI source parameters were set as follows: source temperature of 500°C, ion spray voltage of 5500 V (positive) and −4500 V (negative), ion source gas I (GSI), gas II (GSII), and curtain gas (CUR) set at 55, 60 and 25.0 psi, respectively, and high collision gas (CAD). Instrument tuning and mass calibration were performed using 10 and 100 μmol/L polypropylene glycol solutions in QQQ and LIT modes, respectively. A specific set of multiple reaction monitoring transitions was monitored for each period based on the eluted metabolites. Further analyses, including PCA, HCA, PAA, selection of differentially abundant metabolites and volcano plots, were conducted as described previously.

### 2.7 Protein docking

Crystal structures of irisin (PDB ID 4LSD) and HMOX1 (PDB ID 6EAH) were obtained from the RCSB Protein Data Bank (https://www.rcsb.org/). These structures were then submitted to ClusPro (https://cluspro.org/home.php) for protein–protein docking. After clustering and minimization, various lists of protein–protein complexes were generated, including those favoured by electrostatic interactions, van der Waals forces and electrostatic interactions. For comprehensive analysis, the first prediction from the balanced rank list was selected. Next, LigPlot+2.2.4 was used to identify functional residues involved in hydrogen bonding interactions, salt bridges and hydrophobic interactions. PyMol 2.2.0 was utilized to visualize the protein–protein docking conformation. In LigPlot+2.2.4, irisin was labelled as A and HMOX1 as B. In PyMol, irisin was represented as a slate cartoon, HMOX1 as a cyan cartoon, and their binding sites were displayed as a series of pink sticks. Moreover, the Prodigy tool (https://bianca.science.uu.nl/prodigy/) was employed to calculate the binding energy of the protein–protein complex.

### 2.8 Cell culture

C2C12 myoblasts and MC3T3‐E1 subclone 14 preosteoblast cells were purchased from Procell Life Science and Technology (Wuhan, China). C2C12 myoblasts were cultured in 10% FBS/DMEM and differentiated in 2% horse serum/DMEM. On the first day of differentiation, C2C12 myoblasts were treated with arachidonic acid (AA), DHA and PA (0.01 mM) for 5 days and then collected for further analysis. MC3T3‐E1 preosteoblasts were cultured in 10% FBS/α‐MEM, treated with 0.1 μg/mL recombinant irisin for 48 h, and then collected for further analysis. MC3T3‐E1 preosteoblast cells were differentiated with osteogenic medium (100 nM dexamethasone, 50 μM ascorbic acid and 10 mM β‐glycerophosphate) for 7 or 21 days and then collected for ALP and ARS staining.

### 2.9 siRNA and plasmid transfection

Cell transfections were conducted following the manufacturer's instructions using Lipofectamine® 2000 (Invitrogen, Carlsbad, CA, USA). FNDC5 and Cav1 were separately cloned and inserted into plasmids (GeneChem, Shanghai, China) to overexpress FNDC5 and Cav1 in myoblasts and osteoblasts. In addition, siRNAs against FNDC5, Cav1, HMOX1 and Fpn were transfected into osteoblasts to knock down the expression of the corresponding proteins. The sequences of all plasmids and siRNAs are listed in Table S1.

### 2.10 Immunohistochemical (IHC), immunofluorescence (IHF) stainings and Goldner's trichrome staining

Bone and skeletal muscle tissues were fixed in 4% paraformaldehyde for 48 h, and stored in 70% ethanol before being embedded in paraffin by an embedding machine (Junjie, Wuhan, JB‐P5) and sectioned at 4 μm using a pathology slicer (Leica Shanghai, RM2016). The bone tissue was decalcified prior to being embedded in paraffin. The prepared slides were stored at room temperature and protected from light. Paraffin sections of muscle tissues were used for immunohistochemistry (IHC) of FNDC5 and PGC1α, while paraffin sections of bone tissues were used for immunofluorescence (IHF) of FNDC5, Cav1 and AMPKα. Goldner's trichrome staining was also performed to observe mineralization of bone tissues in paraffin sections.

#### 2.10.1 IHC

Sections were dewaxed in environmentally friendly dewaxing solution (Servicebio, G1128) and rehydrated in successive ethanol baths. Antigen retrieval was performed using the corresponding antigen repair solution: citric acid antigen repair solution (pH 6.0) (Servicebio, G1202) for FNDC5 and EDTA antigen repair solution (pH 9.0) (Servicebio G1203) for PGC1α. After blocking endogenous peroxidase with 3% hydrogen peroxide solution and serum closure with 3% BSA, sections were stained with primary antibodies against FNDC5 (1:400, Proteintech, 23995‐1‐AP) and PGC1α (1:200, Proteintech, 66369‐1‐Ig) at 4°C overnight. Slides were washed with PBS and then stained with the corresponding secondary antibodies at room temperature for 50 min: goat anti‐rabbit IgG (HRP) (1:500, Servicebio, GB23303) for FNDC5 and goat anti‐mouse IgG (HRP) (1:500, Servicebio, GB23301) for PGC1α. Slides were then placed in freshly prepared DAB colour‐developing solution until the positive colour (brown and yellow) was observed under the microscope. The sections were rinsed with tap water to terminate colour development. After nuclear restaining with haematoxylin, the slides were finally dewatered and sealed.

#### 2.10.2 IHF

The dewaxing, rehydration and antigen retrieval of the paraffin sections were performed as described above. Slides were incubated with a primary antibody against AMPKα (1:200, Proteintech, 10929‐2‐AP) overnight at 4°C. Slides were washed in PBS and then incubated in goat anti‐rabbit IgG (CY3) (1:300, Servicebio, GB21303) at room temperature for 50 min. After that, the slides were stained with DAPI for nuclear staining. Slides were then treated with Tissue Autofluorescence Quencher (Servicebio, G1221) to quench tissue autofluorescence and sealed with antifade mounting medium (Servicebio, G1401). Fluorescent homologous double labelling staining of paraffin sections with FNDC5 and Cav1 was performed. Slides were first incubated in primary antibody against FNDC5 and then goat anti‐rabbit IgG (HRP) (1:500, Servicebio, GB23301), which were then treated with fluorescein isothiocyanate (FITC)‐Tyramide (Servicebio, G1222). After antigen retrieval, the slides were incubated with a primary antibody against Cav1 and then goat anti‐rabbit IgG (CY3) (1:300, Servicebio, GB21303). Images were acquired using specific excitation and emission wavelengths for each fluorophore. DAPI excitation wavelength: 330–380 nm, emission wavelength: 420 nm. FITC excitation wavelength: 492 nm, emission wavelength: 520 nm. CY3 excitation wavelength: 510–560 nm, emission wavelength: 590 nm.

#### 2.10.3 Goldner's trichrome staining

Goldner's trichrome staining was performed according to the manufacturer's instructions (Servicebio, G1064). Briefly, Goldner Solution A and Goldner Solution B were mixed in equal volumes according to the required amount. After dewaxing and rehydration, the sections were stained in the mixture for 20 min, followed by rinsing with tap water. Then, they were rapidly differentiated in 1% hydrochloric acid alcohol for 2 s, rinsed with running tap water, and washed with distilled water. The sections were then immersed in Goldner Solutions C, D, C and E for 5 min, with intermittent rinses in 0.2% acetic acid. Slides were then dehydrated in ethanol for 5 min, cleared in xylene for 5 min, and mounted with neutral resin.

### 2.11 Western blot analysis

To prepare the osteoblasts, osteoblasts were initially treated with radioimmunoprecipitation assay (RIPA) lysate and phenylmethanesulfonyl fluoride (PMSF). Subsequently, the osteoblasts were fully lysed using ultrasound, and the lysates were kept on ice for 30 min. After centrifugation at 4°C for 30 min, the supernatant was transferred to a new 1.5‐mL eppendorf (EP) tube for bicinchoninic acid assay (BCA) protein quantification (Beyotime, P0010) Next, 15 μg of protein was subjected to gel electrophoresis and transferred onto a polyvinylidene difluoride membrane. The membrane was then sealed in packaging at room temperature for 2 h, incubated with antibodies (primary antibody at 4°C overnight, and secondary antibody at room temperature for 2 h), and finally exposed for observation.

### 2.12 GST pulldown

The glutathione S‐transferase (GST) pulldown assay was performed as described previously (Chauhan et al., 2016). Briefly, GST‐irisin recombinant protein was expressed in *E. coli*. GST beads were washed and balanced in binding buffer (140 mM NaCl, 2.7 mM KCl, 10 mM Na_2_HPO_4_, and 1.8 mM KH_2_PO_4_, pH 7.4). GST‐irisin was extracted and added to prepared GST beads. After inversion and mixing, the mixture was incubated at room temperature for 30 min. The bead‐protein complex was then isolated on a magnetic separator. Target protein was added to the bead‐protein complex and incubated at room temperature for 30 min. GST protein was used as a negative control. The tube was placed on a magnetic separator, and the supernatant was removed. After washing in binding buffer, elution buffer (50 mM Tris–HCl, 10 mM reduced glutathione, pH 8.0) was added to the complex and isolated on a magnetic separator to acquire an irisin‐target protein mixture, which was stored at −80°C and analysed via WB analysis and mass spectrometry.

### 2.13 Coimmunoprecipitation

Coimmunoprecipitation (CoIP) was performed according to the manufacturer's instructions (Proteintech, PK10007). Briefly, cells were lysed with CoIP lysis buffer (Proteintech, PR20037), and whole‐cell lysates were used for CoIP. Common protease inhibitor cocktail (Proteintech, PR20032) and phosphatase protein inhibitor were added to CoIP lysis buffer (Proteintech, PR20015). The total protein concentration of the lysate was determined using BCA. Lysate with 3 mg of protein was added to a spin column with 4 μg of primary antibody and 300 μL of incubation buffer. Samples were incubated overnight with gentle rotation at 4°C. Samples were then washed with washing buffer and eluted with elution buffer, and the eluted products were boiled and stored at −80°C, which were used for WB analysis.

### 2.14 Cell viability assay

Cell viability was determined using the Cell Counting Kit‐8 (CCK‐8, Dojindo) according to established protocols. Briefly, osteoblasts were seeded at a density of 1 × 104 cells/well in 96‐well plates. After 24 h of treatment with PUFAs or irisin, the cells were incubated with 10 μL of CCK‐8 reagent (diluted in 100 μL medium/well) for 1 h at 37°C in a 5% CO_2_ incubator. The absorbance at 450 nm was measured to assess cell viability.

### 2.15 Determination of ROS, lipid peroxidation and iron

Cells were seeded on six‐well plates and incubated overnight. According to the manufacturer's instructions, cells were treated with DCFH‐DA (Beyotime, S0033S), C11‐BODIPY 581/591 (Invitrogen) and FerroOrange (Cell Signaling Technology, 36104S) to detect reactive oxygen species (ROS), lipid peroxidation and iron, respectively. After incubation in the incubator, cells were digested via pancreatic enzymes and analysed by flow cytometry.

### 2.16 Isolation and identification of exosomes

Exosomes were isolated from the supernatant of myotubes via ultracentrifugation. Supernatants were first centrifuged at 2000 ***g*** at 4°C for 30 min and then transferred into a new centrifuge tube to centrifuge again at 10,000 ***g*** at 4°C for 45 min to remove large vesicles. The supernatant was collected and filtered through a 0.45 μm filter membrane to collect the filtrate. The filtrate was transferred to a new centrifuge tube and centrifuged at 100,000 ***g*** at 4°C for 70 min. The supernatant was removed, and the pellet was collected and resuspended in 10 mL of precooled PBS, which was centrifuged again at 100,000 ***g*** at 4°C for 70 min. The supernatant was removed again to collect exosomes and suspended in 150 μL of precooled PBS. Ten microlitres of exosomes was removed for transmission electron microscopy (TEM) and imaged at 100 kV. Ten microlitres of exosomes was removed and diluted to 30 μL for nanoparticle tracking analysis (NTA) to determine the particle size of exosomes. Exosomes (60 μL) were removed for protein quantification. The remaining exosomes were stored at −80°C for further analysis.

### 2.17 Exosome tracing experiment

The exosome tracing experiment was carried out following the manufacturer's instructions for EvLINK 505 (Cat. EL012100200) and CellLINK 555. Briefly, 5 μL of EvLINK 505 probe was added to 200 μg of exosomes and incubated at room temperature for 30 min. Fluorescently labelled exosomes were obtained via ultrafiltration using 100 kD ultrafiltration membranes (Millipore, UFC510096) and then added to osteoblasts in a 24‐well plate at a density of 3 × 10^4^ cells/well. After 24 h of incubation, the cell culture medium was discarded, and the cells were washed two to three times with Dulbecco's phosphate‐buffered saline DPBS. Then, 5 μL of CellLINK 555 probe was added to osteoblasts to label the cell membrane. Labelled exosomes (Exmax = 505 nm, Emmax = 535 nm) and the cell membrane of osteoblasts (Exmax = 555 nm, Emmax = 585 nm) were observed under transmission electron microscopy (Hitachi, HT7800).

### 2.18 Statistical analysis

Data are presented as means ± SD. Statistical analysis was conducted using Student's *t* test or one‐way analysis of variance. The chi‐square test was employed to assess relationships between qualitative variables. Kaplan–Meier methodology was used to estimate the probability of survival, and differences were evaluated using the log‐rank test. A *p* < 0.05 was considered statistically significant.

## 3 RESULTS

### 3.1 Serum levels of irisin were reduced in postmenopausal patients and could be rescued by exercise

**FIGURE 1:**
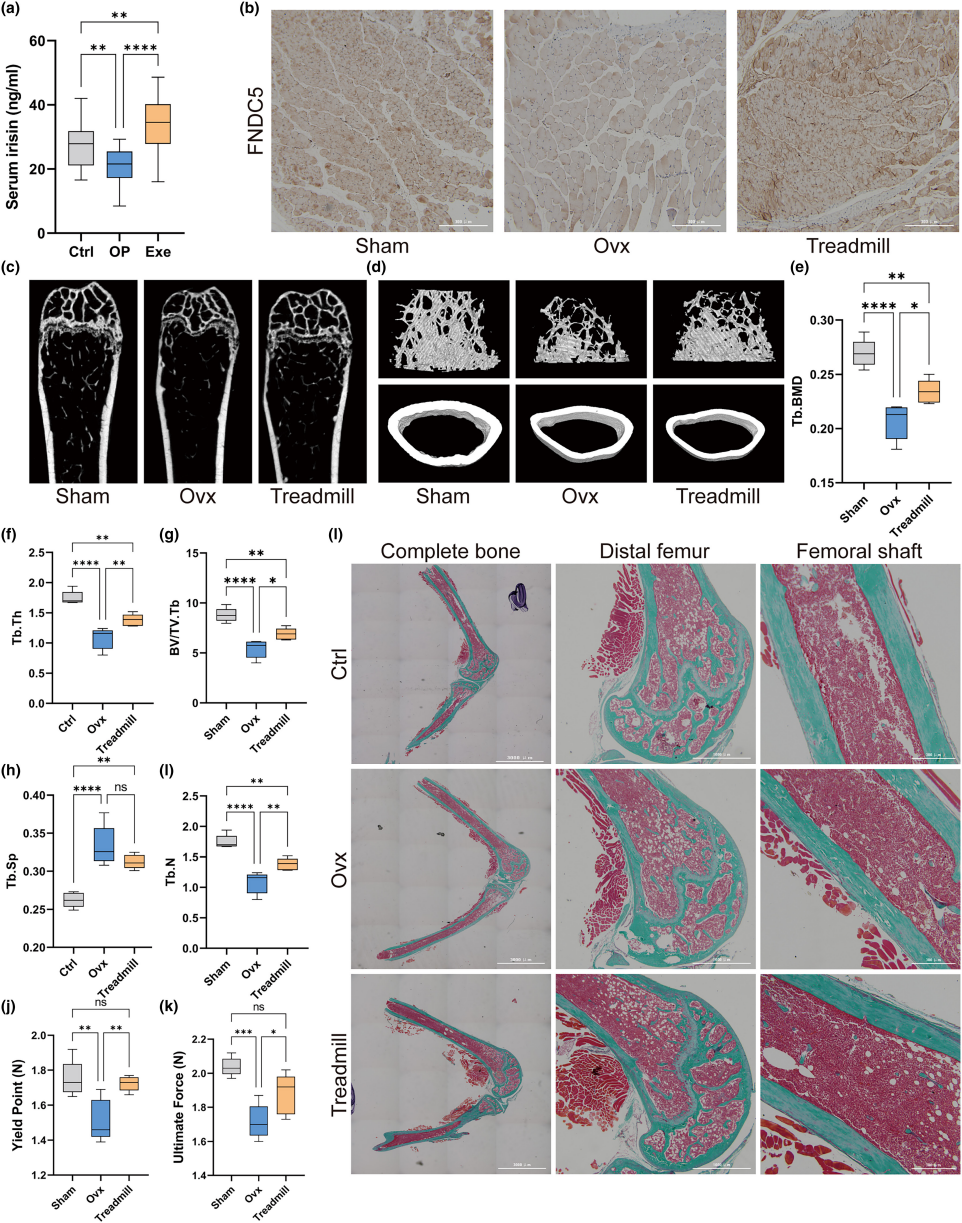
Serum levels of irisin were reduced in postmenopausal patients and could be rescued by exercise. (a) Serum levels of irisin in participants in the control (Ctrl) group, osteoporosis (OP) group, and exercise (Exe) group. (b) Representative images of FNDC5 IHC in skeletal muscle sections. (c) Micro‐CT of the distal femur of mice in the three groups. (d) Three‐dimensional reconstructed micro‐CT images of the femur. Upper panels: trabecular bone; lower panels: cortical bone. (e–i) Femoral trabecular bone mass was assessed by micro‐CT, including the trabecular bone mineral density (Tb.BMD) (e), the trabecular thickness (Tb.Th) (f), the ratio of the bone volume to the total volume (BV/TV) (g), the trabecular separation (Tb.Sp) (h) and the number of trabeculae (Tb.N) (*I*) (*n* = 5 per group). (j, k) Biomechanical analysis of the femur using a 3‐point bending test, including the yield point (j) and ultimate force (k) (*n* = 5 per group). (l) Representative Goldner's trichrome–stained sections of femurs in three groups. Left panels: complete bone (scale bar, 3000 μm), middle panels: distal femur (scale bar, 1000 μm), and right panels: femoral shaft (scale bar, 300 μm). OVX, bilaterally ovariectomized; ns *p* > 0.05, *p < 0.05, **p < 0.01, ***p < 0.001, ****p < 0.0001 (*n* = 3).

Ninety participants were included in this study and were further divided into three groups based on BMD: control group (ctrl), postmenopausal group (OP), and exercise group (Exe). Serum levels of irisin were lower in the OP group and were rescued by long‐term exercise (Figure 1a). For further evaluation of the effect of exercise on BMD, Ovx mice were subjected to 10 weeks of treadmill exercise. The IHC results revealed that FNDC5 was downregulated in the ovariectomized mice, while the 10‐week treadmill exercise significantly upregulated the expression of FNDC5 in skeletal muscles (Figure 1b). In the micro‐CT analysis for BMD and microstructure, Ovx mice exhibited decreased trabecular BMD and bone parameters (Tb.Th, BV/TV.Tb and Tb.SP), while trabecular BMD and bone parameters (Tb.Th and BV/TV.Tb) of the 10‐week treadmill‐exercised mice were significantly increased compared to those of the Ovx mice (Figure 1c–i). We also performed three‐point bending tests in the femurs of each group to evaluate bone strength at a biomechanical level. The yield point reflects the general integrity of the bone, while the ultimate force reflects the general integrity of the bone. The yield point and ultimate force were both decreased in the Ovx group and improved in the treadmill group (Figure 1j,k). Goldner's trichrome staining also confirmed the same results, demonstrating decreased bone mineralization and destroyed bone structure in the Ovx group, which was improved in the treadmill group (Figure 1l).

### 3.2 Ten‐week treadmill exercise ameliorates ovariectomy‐induced metabolomic alterations in skeletal muscle

**FIGURE 2:**
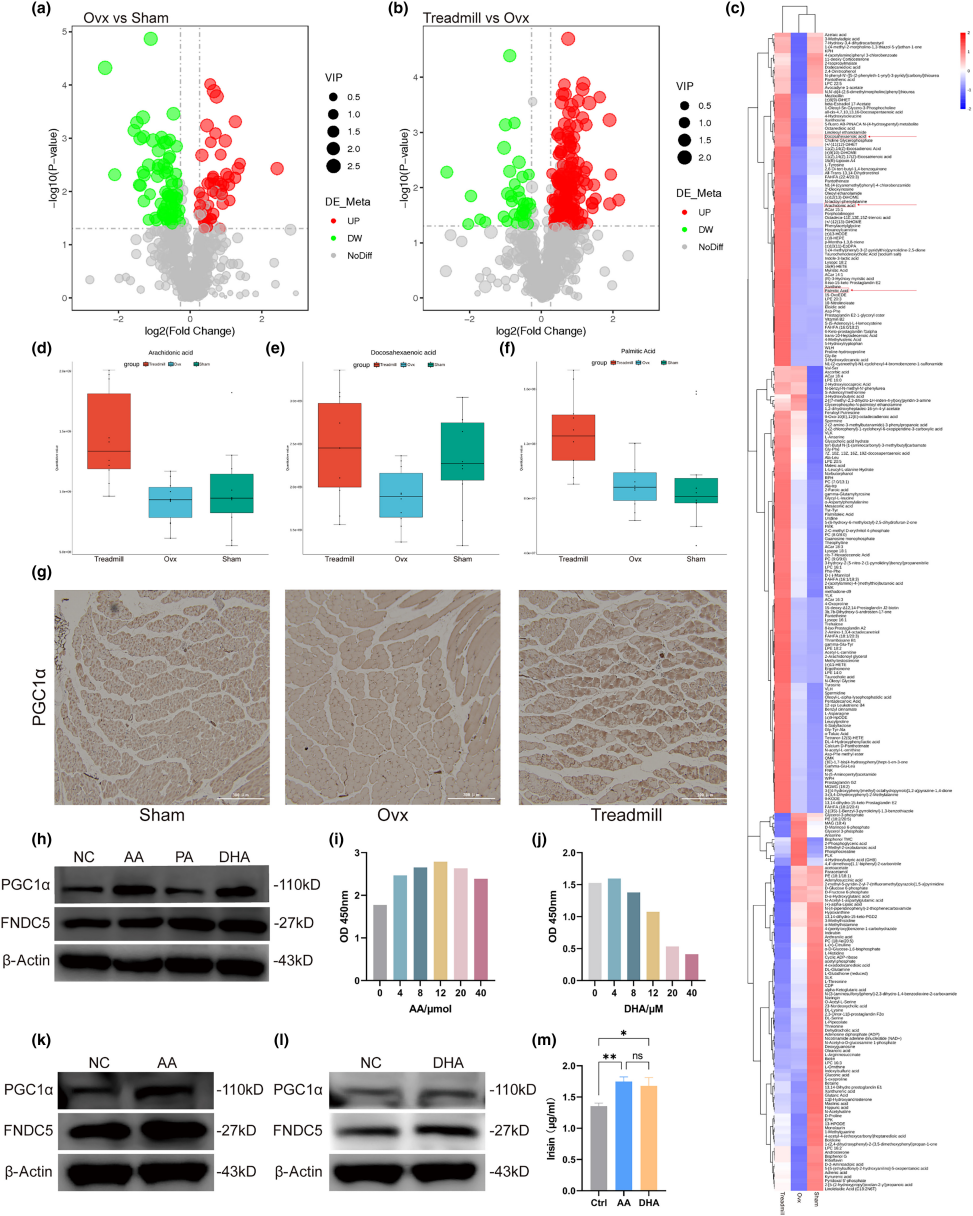
Ten‐week treadmill exercise ameliorates ovariectomy‐induced metabolomic alterations in skeletal muscle. (a,b) Volcano plots of differentially abundant metabolites in skeletal muscles between the Ovx group and Sham group (a) and the treadmill group and Ovx group (b). (c) Clustering heatmap for differentially abundant metabolites among the three groups. The red box with arrows represents polyunsaturated fatty acids (AA, DHA and PA). (d–f) Quantitative values of AA (d), DHA (e), and PA (f) are displayed in the boxplots. (g) Representative images of PGC1α IHC in skeletal muscle sections. Scale bar, 300 μm. (h) Quantification of IHC in (g). (i) Immunoblotting showed the expression of PGC1α and FNDC5 in myotubes treated with AA, DHA and PA for 5 days at 0.01 mM. (j) Quantification of WB in (i). (k, l) Cell viability was analysed with CCK‐8 analysis to determine the effects of AA and DHA on myotubes. (m) Immunoblotting showed the expression of PGC1α and FNDC5 in myotubes treated with AA (12 μM). (n) Quantification of WB in (m). (o) Immunoblotting showed the expression of PGC1α and FNDC5 in myotubes treated with DHA (4 μM). (p) Quantification of WB in (o). (q) ELISA was used to detect concentrations of irisin in the medium of myotubes treated with AA (12 μM) and DHA (4 μM) were measured by ELISAs. AA, arachidonic acid; DHA, docosahexaenoic acid; OVX, bilaterally ovariectomized; PA, palmitic acid; ns *p* > 0.05, *p < 0.05, **p < 0.01, ***p < 0.001, ****p < 0.0001 (*n* = 3).

To further interrogate the role of exercise in regulating expression of FNDC5 in skeletal muscle and BMD among Ovx mice, we used untargeted metabolomics to identify exercises‐related metabolic alterations. To select discriminating metabolites between each pair of groups, we build volcano plots based on the PLS‐DA models (Figure 2a,b). A total of 726 metabolites were identified in each group. A total of 126 significantly differentially abundant metabolites were identified between the Ovx group and Sham group, with 50 increased and 76 decreased. Additionally, 199 significantly differentially abundant metabolites were identified between the Ovx group and Exe group, with 163 increased and 36 decreased. We also made a clustering heatmap among the Sham group, Ovx group, and Exe group to further provide a global view of the metabolite levels across the three groups (Figure 2c). We demonstrated that polyunsaturated fatty acids (PUFAs) can upregulate the expression of the transcriptional coactivator PPAR‐γ coactivator‐1 α (PGC1α) in skeletal muscle cells, while PGC1α could stimulate the expression of FNDC5 (Boström et al., 2012; Rodriguez‐Cruz et al., 2006; Staiger et al., 2005). Our studies also indicated that AA and docosahexaenoic acid (DHA) were decreased in the Ovx group and were significantly increased in the treadmill group (Figure 2d,e). In addition, palmitic acid (PA) increased in the Exe group, although no differences were observed between the Ovx group and Sham group (Figure 2f).

To further determine whether exercise could increase the expression of PGC1α and FNDC5 through the regulation of PUFAs, we analysed the expression of PGC1α in skeletal muscles by IHC. The expression of PGC1α was downregulated in the Ovx group but was restored in the Exe group (Figure 2g,h). We next examined whether PUFAs (AA, DHA and PA) could increase the expression of PGC1α and FNDC5 in vitro. To this end, myoblasts were first differentiated into myotubes and treated with the same concentrations of AA, DHA and PA (10 μM). WB analysis revealed that PGC1α and FNDC5 were upregulated after treatment with AA and DHA, while PA had no effects on the expression of PGC1α and FNDC5 (Figure 2i,j). CCK‐8 assays were then used to determine the optimal drug concentration of AA and DHA. As expected, cell viability was increased with increasing concentrations of AA from 0 to 12 μM and decreased from 12 to 40 μM (Figure 2k). The result was similar for DHA, with increasing cell viability from 0 to 4 μM and decreasing cell viability from 4 to 40 μM (Figure 2l). WB analysis also revealed increased expression of PGC1α and FNDC5 with optimal concentrations of AA (12 μM) and DHA (4 μM) (Figure 2m,p). ELISAs also confirmed that the concentrations of irisin in the culture media of myoblasts were increased after treatment with AA (12 μM) and DHA (4 μM) (Figure 2q).

Meanwhile, we also conducted the untargeted metabolomics sequencing of skeletal muscles obtained from mice in OVX group and OVX group treated with irisin to explore the relationship between treadmill and irisin on the metabolomic alteration of skeletal muscles in OVX mice. PCA exhibited differential metabolites of IRI group and OVX group are well distinguished (Figure S1a). A total of 210 differential metabolites were identified with 154 upregulated and 56 downregulated, which were displayed as volcano plots (Figure S1b). Similar to Exe group, PUFAs including AA, DHA and PA were also identified between differential metabolites between IRI group and OVX group (Figure S1c). Lollipop Chart of differential metabolites exhibited top 20 upregulated and top 20 downregulated differential metabolites, with PA also included (Figure S1d). KEGG bubble map of differential metabolites was further performed to confirm the enriched pathways due to irisin. Interestingly, functional pathways of fatty metabolism were abundantly enriched, including biosynthesis of unsaturated fatty acids, fatty acid metabolism and fatty acid degradation, et al, which are highly similar to Exe group (Figure S1e). In addition, Ferroptosis was also enriched in the KEGG enrichment analysis, which was similar to the results validated in osteoblasts which will be discussed later in the paper. Based on the above results, we found that exercise and irisin have a very similar impact on the metabolic alterations in skeletal muscle, demonstrating that irisin plays a key role in the bone‐protective effects exerted by exercise.

### 3.3 Irisin can increase the bone mass of Ovx mice and promote the differentiation of osteoblasts

**FIGURE 3:**
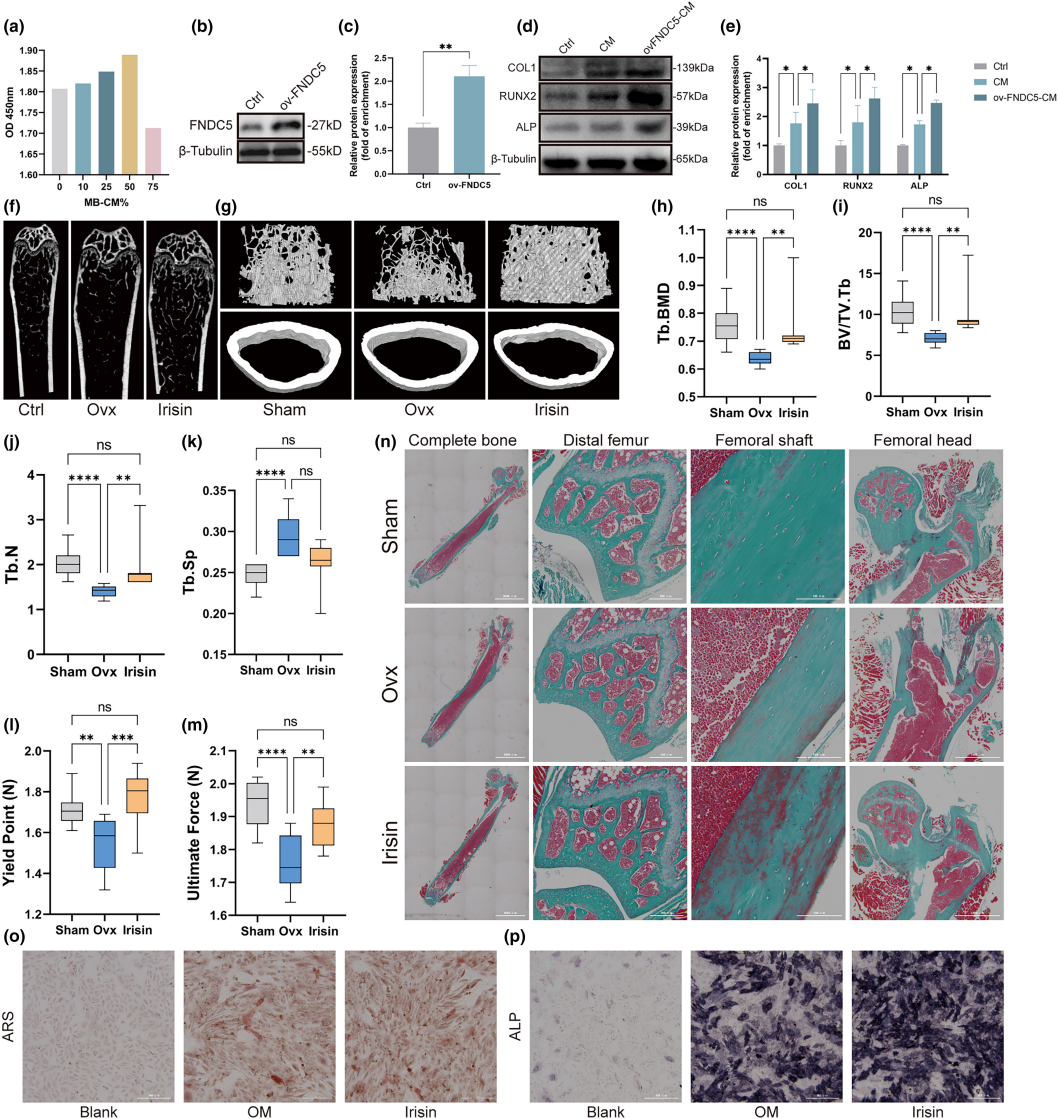
Irisin increased the bone mass of Ovx mice and promoted the differentiation of osteoblasts. (a) Cell viability of osteoblasts treated with different concentrations of conditioned medium (CM) from myotubes. (b) Representative western blot analysis of FNDC5 expression indicated that FNDC5 was successfully overexpressed in myotubes. (c) Quantification of WB in (b). (d) Immunoblotting showed the expression of COL1, RUNX2 and ALP in osteoblasts treated with CM from myotubes transfected with or without FNDC5 plasmid. (e) Quantification of WB in (d). (f) Micro‐CT of the distal femur of mice in the three groups. (g) Representative 3D reconstructed micro‐CT images of the femur. Upper panels: trabecular bone; lower panels: cortical bone. (h–k) Trabecular bone microarchitecture of femurs showing Tb.BMD (h), BV/TV (i), Tb.N (j) and Tb.Sp (k) of three groups (*n* = 10 per group). (l, m) Biomechanical analysis of the femur using a 3‐point bending test, including the yield point (l) and ultimate force (m) (*n* = 10 per group). (n) Representative Goldner's trichrome–stained sections of femurs in three groups. First column, complete bone (scale bar, 3000 μm); second column, distal femur (scale bar, 300 μm); third column, femoral shaft (scale bar, 100 μm); fourth column, femoral head (scale bar, 1000 μm). (o) Alizarin red staining of osteoblasts cultured in osteogenic medium (OM) treated with irisin (irisin group) or without irisin (OM group) compared with osteoblasts cultured in basal medium (blank group) was performed on the seventh day of differentiation. Scale bar, 300 μm. (p) ALP staining of osteoblasts of three groups on the 21st day of differentiation. OVX, bilaterally ovariectomized; ARS, Alizarin red staining; ALP, Alkaline phosphatase staining; Scale bar, 300 μm. ns *p* > 0.05, *p < 0.05, **p < 0.01, ***p < 0.001, ****p < 0.0001 (*n* = 3).

To determine if irisin secreted by myotubes could promote the cell viability of osteoblasts, we cultured osteoblasts with different concentrations of conditioned media (CM) of myotubes and assessed them via CCK‐8 assays. The CCK‐8 assay results showed that a final concentration of 50% CM showed the strongest promotion of cell viability (Figure 3a). We then transfected the FNDC5 plasmid into myotubes and collected their supernatants. Successful transfection and expression were verified by WB (Figure 3b,c). Osteoblasts treated with CM from myotubes exhibited increased levels of osteogenic markers (ALP, RUNX2 and COL1), while osteoblasts treated with CM from myotubes transfected with FNDC5 plasmids exhibited higher levels of osteogenic markers (Figure 3d,e).

To determine whether irisin could ameliorate osteoporosis‐induced bone loss, we intraperitoneally injected irisin (100 μg/kg) into Ovx mice once a week for 4 weeks. Femoral trabecular bone mass assessed by micro‐CT analysis revealed obvious bone loss in the Ovx group, and this change was significantly rescued in the irisin group (Figure 3f,g). As expected, compared to Sham group, Tb.BMD, BV/TV.Tb and TB.N were significantly decreased in OVX group, while OVX group also exhibited a significant increase in Tb.Sp. Meanwhile, decreased Tb.BMD, BV/TV.Tb and Tb.N were restored significantly in the irisin group (Figure 3h,j). However, different from Exe group mentioned above, there was no significant difference in Tb.Sp (Figure 3k). Bone strength, including yield point and ultimate force, was also improved during the three‐point bending test on the femurs, collectively indicating the protective effect of irisin on bone loss outcomes (Figure 3l,m). Moreover, Goldner's trichrome staining confirmed that mineralized bone was reduced significantly in the Ovx group, and this change was improved in the irisin group. Additionally, a large amount of newly formed bone was observed in the cortical bone of the irisin group, indicating an increased capacity for bone formation induced by irisin (Figure 3n). To determine the capacity of irisin to promote osteogenic differentiation in vitro, we seeded osteoblasts into 6‐well plates and then induced them to differentiate, accompanied by irisin treatment (0.1 μg/mL). ALP staining and ARS staining were performed at days 7 and 21 of osteogenic differentiation to monitor osteogenesis. The results of ALP staining and ARS staining showed that irisin significantly facilitated differentiation of osteoblasts (Figure 3o,p).

### 3.4 Cav1 interacts with FNDC5 and AMPKα to activate the AMPK pathway

**FIGURE 4:**
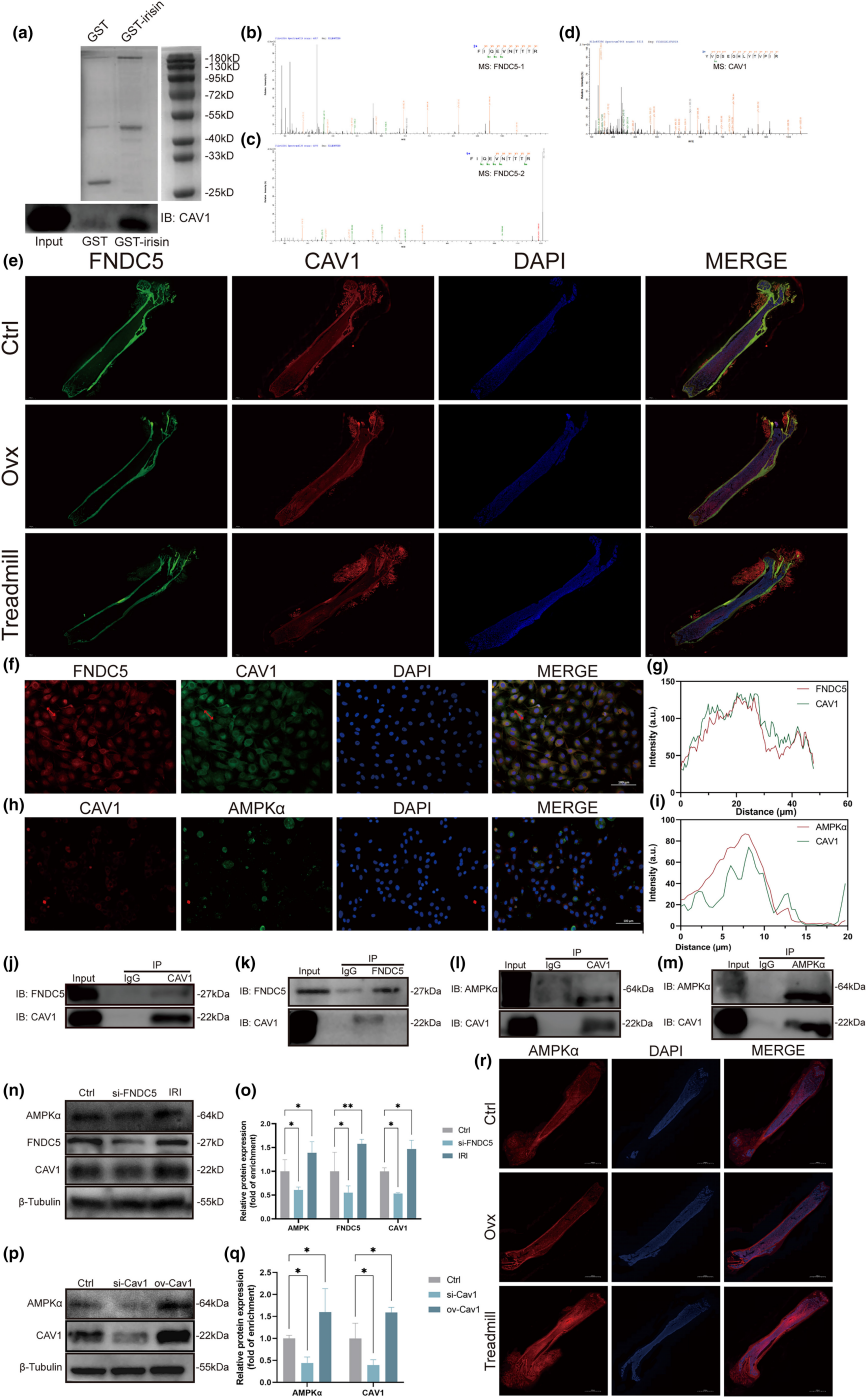
Cav1 interacts with FNDC5 and AMPKα to activate the AMPK pathway. (a) GST pulldown assays of irisin. GST‐irisin was used as bait, while GST served as a negative control (upper panel). Immunoblotting identified the expression of CAV1 in GST‐irisin pulldown products (lower panel). (b, c) Secondary mass spectrometry analysis of GST pulldown products exhibiting specific peptide segments identified as FNDC5. (d) Secondary mass spectrometry analysis of GST pulldown products exhibiting specific peptide segments identified as CAV1. (e) Immunofluorescence staining of femurs from mice in the control group, Ovx group and treadmill group. Sections were stained with antibodies against FNDC5 (green) and CAV1 (red). Nuclei were stained with DAPI (blue). Scale bar, 1000 μm. (f) Colocalization images of FNDC5 (red) and CAV1 (green) in osteoblasts. Nuclei were stained with DAPI (blue). Osteoblasts with red arrows were used for analysis of fluorescence intensity and colocalization by ImageJ. Scale bar, 100 μm. (g) Intensity profile of FNDC5 and Cav1 immunofluorescence. (h) Colocalization images of Cav1 (red) and AMPKα (green) in osteoblasts. Nuclei were stained with DAPI (blue). Osteoblasts with red arrows were used for analysis of fluorescence intensity and colocalization by ImageJ. Scale bar, 100 μm. (i) Intensity profile of FNDC5 and Cav1 immunofluorescence. (j, k) Coimmunoprecipitation (CoIP) of FNDC5 and CAV1 in osteoblasts. (l, m) CoIP of CAV1 and AMPKα in osteoblasts. (n) Osteoblasts were transfected with FNDC5‐siRNA and irisin (1 μg/mL). Immunoblotting showed the expression of AMPKα, FNDC5 and CAV1. (o) Quantification of WB in (n). (p) Osteoblasts were transfected with CAV1‐siRNA and CAV1 plasmid. Immunoblotting showed the expression of AMPKα and CAV1. (q) Quantification of WB in (p). (r) IF staining of femurs to evaluate the expression of AMPKα in femur tissues. OVX, bilaterally ovariectomized; IRI, irisin; ns *p* > 0.05, *p < 0.05, **p < 0.01 (*n* = 3).

We next determined the mechanism by which irisin could rescue bone loss induced by Ovx and promote osteoblast differentiation. A GST pulldown assay was performed as described previously to identify potential proteins binding with irisin. The band corresponding to GST‐irisin was cut out and analysed by second mass spectrometry. Cav1 was identified to bind to irisin via mass spectrometry and was also verified through WB (Figure 4a). Figure 4b,c showed the existence of specific peptide segments identified as FNDC5 (Figure 4b,c), while Figure 4d exhibited specific peptide segments of Cav1 (Figure 4d). Immunofluorescence staining of femurs was used to determine the spatial localization of FNDC5 and Cav1 in femur tissues. FNDC5 was more highly expressed in cortical bone, while Cav1 was expressed in both cortical bone and trabecular bone (Figure 4e). To further confirm the subcellular localization of FNDC5 and Cav1, we performed immunofluorescence colocalization analysis, and the data were analysed via ImageJ. As shown in Figure 4f, FNDC5 colocalized with Cav1 in the cytoplasm. The intensity profile of FNDC5 and Cav1 immunofluorescence also indicated their colocalization (Figure 4g). In addition, Cav1 was shown to interact with AMPKα and activate the AMPK pathway in osteoblasts (Tang et al., 2022). We thus also performed immunofluorescence colocalization analysis to determine whether Cav1 could interact with AMPKα in osteoblasts. Cav1 and AMPKα also overlapped in the cytoplasm, which was verified by the intensity profile (Figure 4h,i). Next, we performed CoIP experiments to further identify endogenous interactions among these proteins. The CoIP assays revealed that FNDC5 interacted with Cav1 (Figure 4j,k). Additionally, the interaction between Cav1 and AMPKα was verified through CoIP assays (Figure 4l,m). We further investigated whether there are regulatory relationships among FNDC5, Cav1 and AMPKα. WB results revealed that FNDC5/irisin could regulate the expression of Cav1 and AMPKα. Osteoblasts transfected with FNDC5‐siRNA exhibited decreased levels of Cav1 and AMPKα, while high levels of Cav1 and AMPKα were noted in osteoblasts treated with irisin (Figure 4n,o). Additionally, we transfected osteoblasts with Cav1‐siRNA and Cav1‐plasmid. Decreased expression of Cav1 and AMPKα was observed in the osteoblasts transfected with Cav1‐siRNA, while increased expression of Cav1 and AMPKα was observed in the osteoblasts transfected with Cav1‐plasmid (Figure 4P,Q). We also performed IF staining of femurs to confirm whether treadmill exercise could promote the expression of AMPKα in vivo. The results revealed that AMPKα was expressed in both cortical and cancellous bone, while treadmill exercise upregulated AMPKα expression in femur tissues (Figure 4r).

### 3.5 Irisin can increase serum concentrations of biliverdin by upregulating HMOX1

**FIGURE 5:**
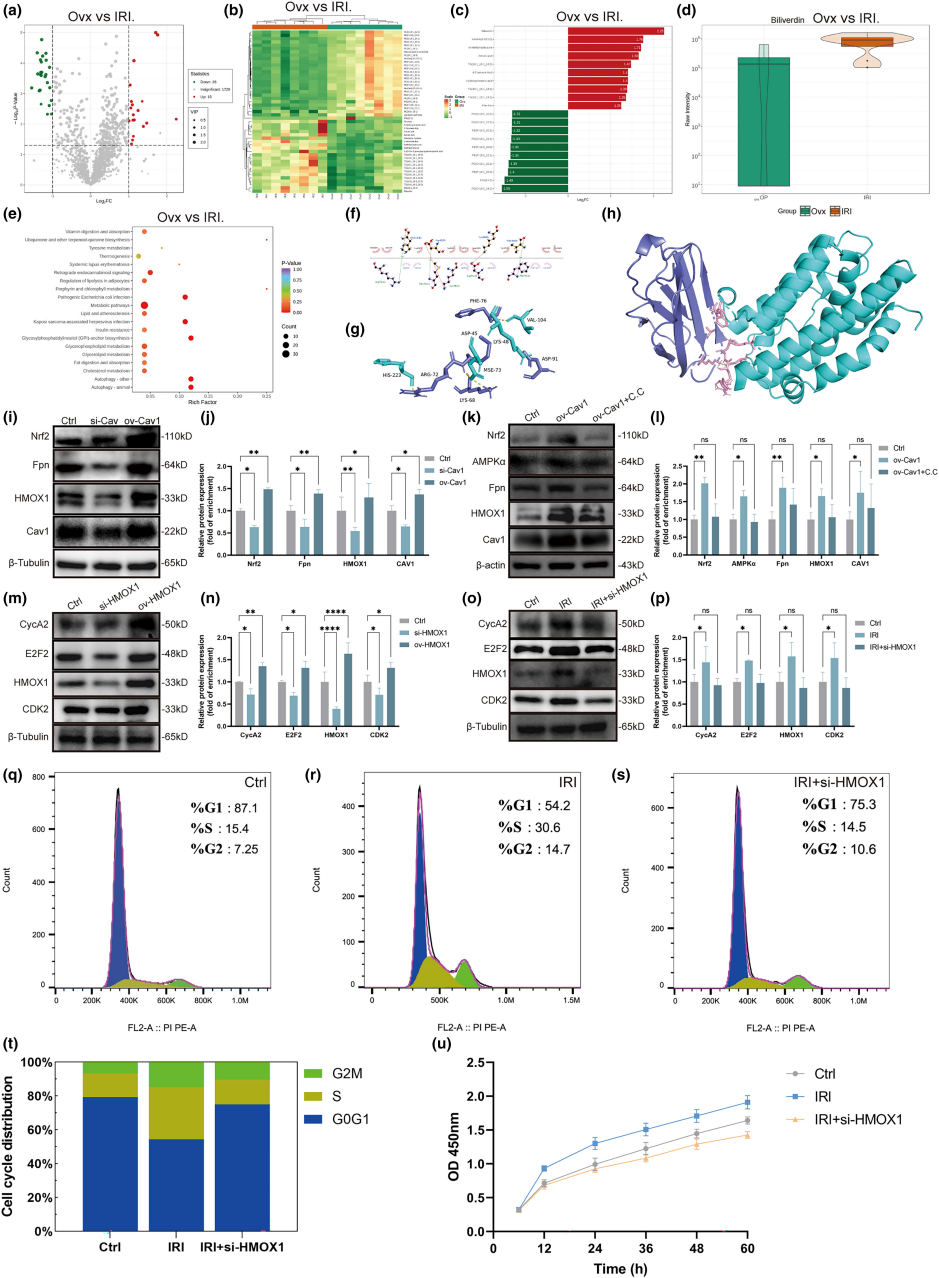
Irisin could regulate the cell cycle and promote the proliferation of osteoblasts via the upregulation of HMOX1. (a) Volcano plots of differentially abundant metabolites in serum between the Ovx group and IRI group. (b) Clustering heatmap for differentially abundant metabolites between the Ovx group and IRI group. (c) Column graph showing the fold change of the top 10 increased and decreased metabolites. (d) Violin plots showing the serum level of biliverdin between the Ovx group and IRI group. (e) KEGG pathway enrichment analysis of differentially abundant metabolites between the Ovx group and IRI group. (f, h) Interaction between irisin and HMOX1. (f) Irisin was labelled as (a), and HMOX1 was labelled as (b). (g) Irisin is shown as cyan sticks, and HMOX1 is shown as slate sticks. (h) Irisin is represented as a slate cartoon, HMOX1 is presented as a cyan cartoon, and their binding sites are displayed as a series of pink sticks. (i) Osteoblasts were transfected with Cav1‐siRNA and Cav1 plasmid. Immunoblotting showed the expression of Nrf2, Fpn, Cav1 and HMOX1 in osteoblasts. (j) Quantification of WB in (i). (k) Osteoblasts were transfected with Cav1 plasmid and further treated with C.C. (1 μM) for 2 h. Immunoblotting showed the expression of Nrf2, AMPKα, Fpn, HMOX1 and Cav1 in osteoblasts. (l) Quantification of WB in (k). (m) Osteoblasts were transfected with HMOX1‐siRNA and HMOX1 plasmid. Immunoblotting showed the expression of CycA2, E2F2, HMOX1 and CDK2 in osteoblasts. (n) Quantification of WB in (m). (o) Osteoblasts were transfected with HMOX1‐siRNA and then treated with irisin (0.1 μg/mL) for 5 days. Immunoblotting showed the expression of CycA2, E2F2, HMOX1 and CDK2 in osteoblasts. (p) Quantification of WB in (o). (q–s) The cell cycle distribution of osteoblasts in the three groups was analysed by flow cytometry. (t) Stacked column graph exhibiting cell cycle distribution in (q–s). (u) A CCK‐8 assay was used to analyse the proliferation of osteoblasts in the three groups. ns *p* > 0.05, *p < 0.05, **p < 0.01, ***p < 0.001, ****p < 0.0001 (*n* = 3).

To explore the downstream molecular mechanism of activation of the AMPKα pathway induced by irisin, we determined serum metabolic profiles based on an ultra‐performance liquid chromatography/tandem mass spectrometry (UPLC–MS/MS) metabolomics approach between Ovx mice with or without irisin treatment. Volcano plots showed significant alterations in serum metabolites due to irisin administration (Figure 5a). A total of 49 significant metabolites were identified, with 22 increased and 27 decreased. These 49 metabolites also exhibited good clustering and reliable differentiation between the Ovx group with and without irisin treatment (Figure 5b). We then generated a column graph showing the fold changes of the top 10 increased and decreased metabolites (Figure 5c). Among these, biliverdin was the most differentially accumulating metabolite (log_2_FC = 2.25). Violin plots also showed the level of biliverdin between the two groups (Figure 5d). KEGG enrichment analysis was performed to confirm the enriched pathways and confirmed 27 enriched pathways (Figure 5e). Biliverdin is a metabolite involved in the haem catabolic pathway. Haem oxygenase‐1 (HMOX1) is the rate‐limiting enzyme that cleaves haem to generate biliverdin, carbon monoxide and ferrous iron, which is known to modulate various cellular functions, including cell proliferation and apoptosis (Haider & Ashraf, 2008).

To confirm whether there are regulatory relationships between irisin and HMOX1, we performed molecular docking to confirm whether irisin could bind directly to HMOX1. Via LigPlot+2.2.4, we identified all functional residues and sorted them by their interactions. In hydrogen bonding interactions, Arg‐72, Met‐73 and Phe‐76 of irisin and His‐223, Asp‐45 and Val‐104 of HMOX1 contributed to the construction. Some of the remaining residues, such as Lys 68 and Asp 91 of irisin and Lys‐48 of HMOX1, were used to construct salt bridges or participated in forming hydrophobic forces (Figure 5f–h). We obtained the exact binding energy and Kd by Prodigy. The binding energy of irisin and HMOX1 was −7.7 kcal/mol, which indicated their poor binding capacity. CoIP assays were further conducted to evaluate the interaction of irisin and HMOX1, indicating that there was weak interaction between irisin and HMOX1 in osteoblasts (Figure S2a,b).

### 3.6 Nrf2 is upregulated, followed by activation of AMPKα and increased expression of HMOX1 and Fpn

We next sought to confirm whether HMOX1 is the downstream target gene of AMPKα pathway activation. Nuclear factor E2‐related factor 2 (Nrf2) is a transcription factor that activates the transcription of various antioxidant genes (Wang et al., 2018). HMOX1 and Fpn are downstream targets of Nrf2 (Grignano et al., 2020; Soares & Hamza, 2016) and were reported to be involved in the regulation of cell fate, such as the cell cycle and iron metabolism (Boylston & Brenner, 2014; Tao et al., 2020). The WB results showed that Cav1 knockdown or overexpression resulted in the inhibition or promotion of Nrf2/HMOX1 expression, respectively (Figure 5i,j). Additionally, the upregulation of Nrf2, HMOX1 and Fpn by Cav1 overexpression was suppressed by the AMPK inhibitor Compound C (C.C) (Figure 5k,l). Therefore, Nrf2 was proven to be upregulated when AMPKα was activated by Cav1, which subsequently induced the transcription of HMOX1 and Fpn.

### 3.7 Irisin can regulate the cell cycle and promote the proliferation of osteoblasts via upregulation of HMOX1

As mentioned above, HMOX1 regulates cell proliferation in multiple cell lines (Fiorito et al., 2019; Piechota‐Polanczyk et al., 2015). In addition, irisin was shown to be involved in the regulation of the cell cycle and cell proliferation (Chen et al., 2022). Therefore, we speculated that irisin might also regulate the cell cycle and promote the proliferation of osteoblasts. In particular, we analysed the effect of HMOX1 on the expression of cycle‐associated proteins (E2F2, CycA2 and CDK2). WB results revealed that the expression of these proteins was reduced after knockdown of HMOX1, while overexpression stimulated their expression (Figure 5m,n). In addition, irisin (IRI) promoted the expression of cycle‐associated proteins, which were significantly suppressed by knockdown of HMOX1 (Figure 5o,p). To further determine the effect of irisin on cell cycle progression, we performed propidium iodide (PI) staining to analyse the cell cycle in osteoblasts treated with irisin. Irisin significantly increased S‐phase and G2/M‐phase cells, and this effect was reversed after transfection with HMOX1‐siRNA (Figure 5q–s). Stacked column graph also exhibited increased proportion of G2/M‐phase and S‐phase due to treatment with irisin, which was rescued when transfected with HMOX1‐siRNA (Figure 5t). Increased numbers of S‐phase and G2/M‐phase cells might indicate enhanced proliferative activity. We next performed a CCK‐8 assay to further determine cell viability. The results showed that irisin significantly promoted the proliferation of osteoblasts, which was also suppressed when cells were transfected with HMOX1‐siRNA (Figure 5u). Taken together, our results showed that irisin could promote osteoblast proliferation by promoting cell cycle progression, which is dependent on HMOX1.

### 3.8 Irisin can inhibit ferroptosis of osteoblasts via upregulation of Fpn

**FIGURE 6:**
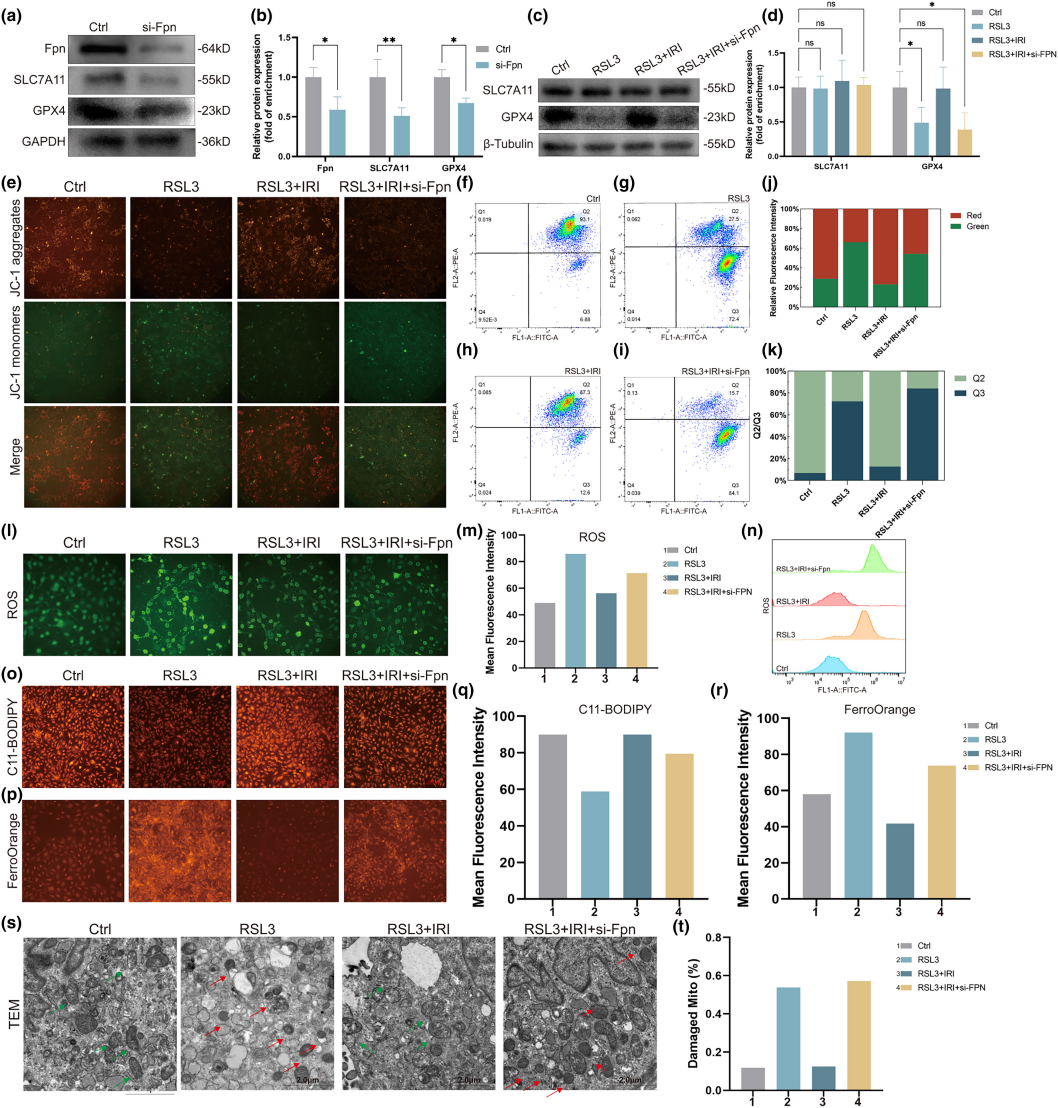
Irisin inhibited ferroptosis of osteoblasts via upregulation of Fpn. (a) Osteoblasts were first transfected with Fpn‐siRNA. Immunoblotting showed the expression of Fpn, SLC7A11 and GPX4 in osteoblasts. (b) Quantification of WB in (a). (c) RSL3 (1 μM) was added to induce ferroptosis of osteoblasts. Irisin (1 μg/mL) was used to suppress ferroptosis. Immunoblotting showed the expression of SLC7A11 and GPX4 in osteoblasts. (d) Quantification of WB in (c). (e) Representative images of JC‐1 staining in osteoblasts. (f–i) Quantitation of JC‐1 fluorescence by flow cytometry in osteoblasts. (j) Stacked column graph of JC‐1 staining via fluorescence images in (e). (k) Stacked column graph of JC‐1 staining via flow cytometry in (f–i). (l) Representative images of ROS staining in osteoblasts. (m) Quantification of ROS fluorescence images in (l). (n) Quantitation of ROS fluorescence by flow cytometry in osteoblasts. (o) Representative images of lipid ROS staining in osteoblasts with C11‐BODIPY. (p) Representative images of intracellular iron staining in osteoblasts with FerroOrange. (q) Quantification of C11‐BODIPY fluorescence images in (o). (r) Quantification of FerroOrange fluorescence images in (p). (s) The microstructure of mitochondria in osteoblasts was observed by TEM. Red arrows indicate mitochondria. Typical morphological changes in mitochondria are distinctive characteristics of ferroptosis, exhibiting mitochondrial shrinkage with condensed mitochondrial membrane densities and loss of mitochondrial ridges. Scale bar, 2 μm. (t) Quantification of damaged mitochondria percentages in (S). IRI, irisin; 1, Ctrl group; 2, RSL3 group; 3, RSL3 + IRI group; 4, RSL3 + IRI + si‐Fpn group; ns *p* > 0.05, *p < 0.05, **p < 0.01 (*n* = 3).

Our study confirmed that Fpn was the downstream target gene of Nrf2, which was responsible for the removal of iron in osteoblasts. It was previously indicated in this study that upregulation or downregulation of Cav1 could lead to increased or decreased expression of Fpn, respectively, while AMPK inhibitor could rescue the expression level of Fpn caused by increased Cav1 (Figure 5i–l). We next explored whether irisin could suppress ferroptosis. Osteoblasts were first transfected with Fpn‐siRNA, and the WB results showed that decreased SLC7A11 and GPX4, which are known to suppress ferroptosis, were accompanied by Fpn knockdown (Figure 6a,b). Thus, Fpn knockdown might induce ferroptosis in osteoblasts, which subsequently decreases the expression of SLC7A11 and GPX4. RSL3 (RAS‐selective lethal 3) is a canonical ferroptosis inducer that inhibits GPX4 (Lang et al., 2019). We next treated osteoblasts with RSL3 and irisin to identify whether irisin could ameliorate ferroptosis induced by RSL3. WB results showed that RSL decreased the expression of GPX4, which was rescued by irisin, while Fpn knockdown significantly suppressed the antiferroptotic effects of irisin (Figure 6c,d). However, different from GPX4, the expression of SLC7A11 was not significantly altered (Figure 6c,d). It might be explained that RSL3 is the inhibitor of GPX4, and thereby changes in SLC7A11 expression levels exhibit relatively low.

To further confirm the role of irisin in inhibiting ferroptosis, we performed JC‐1 staining to detect alterations in the mitochondrial membrane potential (MMP). Our results showed that the ratio of JC‐1 aggregates to JC‐1 monomers decreased in RSL3 cells, indicating that the lower MMP was caused by RSL3‐induced ferroptosis (Figure 6e). Moreover, irisin could increase MMP osteoblasts treated with RSL3, which was then suppressed when cells were transfected with Fpn‐siRNA, suggesting that irisin could restore the decline in MMP due to RSL3‐induced ferroptosis dependent on Fpn (Figure 6e). This finding was also confirmed by flow cytometry analysis to detect the MMP of these groups (Figure 6f–i). These results were also visually exhibited in stacked column graphs (Figure 6j,k). Decreased MMP is frequently accompanied by increased levels of ROS. Moreover, ferroptosis is frequently characterized by the accumulation of lipid peroxides and the production of ROS in mitochondria (Zhou et al., 2020). Therefore, we next added the fluorescent probes DCFH‐DA, FerroOrange and C11‐BODIPY to osteoblasts to measure the levels of intracellular ROS, lipid ROS and ferrous ions, respectively. Fluorescence imaging results showed that osteoblasts treated with RSL3 exhibited bright fluorescence, while irisin attenuated the fluorescence intensity, and this effect was further increased when the cells were transfected with Fpn‐siRNA (Figure 6l,m). This finding was also verified via flow cytometry analysis of ROS (Figure 6n). C11‐BODIPY (581/591) is frequently used to detect lipid ROS. When C11‐BODIPY (581/591) reacts with lipid ROS, the fluorescence emission peak shifts from 590 nm (red) to 510 nm (green). Hence, weak red fluorescence indicates high levels of lipid ROS. Thus, a high level of lipid ROS was observed in osteoblasts treated with RSL3 and was attenuated by irisin (Figure 6o). Similar to the fluorescence results of ROS, osteoblasts transfected with Fpn‐siRNA also exhibited high levels of lipid ROS, despite being treated with irisin (Figure 6p). We also found that irisin attenuated intracellular iron overload caused by RSL3, which was further exacerbated when cells were transfected with Fpn‐siRNA (Figure 6p). These results were further exhibited in bar charts intuitively (Figure 6q,r). Changes in mitochondrial morphology are also a distinctive feature of ferroptosis, which is mainly characterized by membrane thickening, disappearance of mitochondrial cristae, and rupture of mitochondria (Zhang et al., 2020). TEM showed that mitochondria of osteoblasts treated with RSL3 exhibited typical morphological changes of ferroptosis (mitochondrial shrinkage with condensed mitochondrial membrane densities and loss of mitochondrial ridges), which were recovered by irisin and were further aggravated after transfection with Fpn‐siRNA (Figure 6s). It was also confirmed in the bar chart of the damaged mitochondria percentage (Figure 6t).

### 3.9 Exosomes mediate the transportation of FNDC5/irisin from myotubes to osteoblasts

We next sought to confirm whether irisin could be secreted via exosomes and then transported to bone tissues to act on osteoblasts. Myoblasts were first transfected with the FNDC5 expression plasmid and then differentiated into myotubes. Cell supernatants were collected 5 days after differentiation, and the supernatant was taken for subsequent ultracentrifugation to isolate exosomes. TEM showed the typical cup‐shaped morphology of exosomes isolated from both the ctrl group and ov‐FNDC5 group (Figure S3a,b). Further characterization of these exosomes by NTA revealed that both myotube‐derived exosomes and ov‐FNDC5 myotube‐derived exosomes exhibited moderate size. The particle size of myotube‐derived exosomes was 83.7 ± 18.2 nm, while the particle size of ov‐FNDC5 myotube‐derived exosomes was 82.2 ± 18.1 nm (Figure S3c,d). Exosomes were quantified as numbers of exosomes per ml. The concentration of myotube‐derived exosomes was 7.44 × 10^9^ particles/mL, while concentration of ov‐FNDC5 myotube‐derived exosomes was 4.38 × 10^9^ particles/mL (Figure S3e,f). WB analysis was then performed to further identify exosomes, indicating the existence of exosome protein biomarkers (TSG101 and CD9) and the nonexistence of nonexosome markers (calnexin) (Figure S3g). In vitro tracing confirmed that osteoblasts were capable of taking up both myotube‐derived exosomes and ov‐FNDC5 myotube‐derived exosomes (Figure S3h). Therefore, our results confirmed that exosomes could mediate cellular communication between myotubes and osteoblasts.

We next sought to confirm whether irisin exists in myotube‐derived exosomes and subsequently promotes osteoblast differentiation. WB analysis confirmed that FNDC5/irisin exists in myotube‐derived exosomes, which was increased after transfection with FNDC5 expression plasmids (Figure S3i). GW4869 has been described to inhibit exosome release. FNDC5/irisin in exosomes was significantly downregulated after treatment with GW4869, suggesting that increased FNDC5/irisin expression in myotubes could upregulate FNDC5/irisin expression in exosomes (Figure S3i). We then treated osteoblasts with exosomes derived from myotubes and the GW4869 group to determine the effect of myotube‐derived exosomes on osteoblasts. WB analysis revealed that myotube‐derived exosomes could increase the expression of osteogenic markers (COL1, RUNX2 and OCN), while exosomes derived from myotubes treated with GW4869 had no effects on osteoblasts (Figure S3j,k). In order to evaluate the role of FNDC5 levels in myotubes on the osteogenic capac2ity of osteoblasts, exosomes derived from myotubes which are transfected with FNDC5‐plasmid (ovFNDC5 MB‐Exo) were added to osteoblasts. WB analysis showed that upregulation of FNDC5 in myotubes could significantly increase the expression of osteogenic markers in osteoblasts treated with myotube‐derived exosomes (Figure S3l,m). We also performed ALP staining and ARS staining to further determine the prodifferentiation effects of myotube‐derived exosomes. ARS staining showed that myotube‐derived CM could promote the differentiation of osteoblasts, which was strengthened after treatment with CM from ovFNDC5‐myotubes (Figure S3n). CM derived from myotubes treated with GW4869 showed no prodifferentiation effects (Figure S3n). ALP staining revealed similar results (Figure S3o). Therefore, our results revealed that FNDC5/irisin could be secreted via exosomes and then acted on osteoblasts to promote osteogenic differentiation.

## 4 DISCUSSION

Consistently, regular exercise has been shown to be effective in promoting bone health. However, due to the difficulty for postmenopausal women to adhere to long‐term regular exercise, it is crucial to explore alternative therapies that serve as effective substitutes for exercise. Recent studies have shown that skeletal muscle produces various myokines in response to exercise, enabling crosstalk between skeletal muscle and other organs. Irisin, a recently discovered myokine that is produced in response to exercise, plays a crucial role in providing substantial protective effects on bone (Xue et al., 2022). Despite the knowledge that irisin is significantly upregulated during exercise, the mechanisms underlying its upregulation and the process by which it is transported from skeletal muscle to bone are still not fully understood. Our study confirmed for the first time that polyunsaturated fatty acids (AA and DHA), which are increased in skeletal muscle during exercise, could enhance the expression of PGC1α, which then stimulates the transcription of FNDC5 in myotubes and subsequently increases the release of irisin. Furthermore, we found that FNDC5/irisin is trafficked to osteoblasts via exosomes and enters osteoblasts through caveolae‐mediated endocytosis. Our study revealed why irisin is upregulated during exercise and elucidated the mechanisms involved in its transportation to bone. This is also the first study to identify Cav1 as a crucial protein during the endocytosis of FNDC5/irisin, and this molecule also serves as the key downstream effector of FNDC5/irisin in osteoblast.

FNDC5/irisin was shown to exert its effects on distant organs through exosomes. Chi C et al. demonstrated that FNDC5/irisin in circulating EVs released from skeletal muscles could exert a senescence‐delaying effect on vascular smooth muscle cells (Chi et al., 2022). Consistent with a previous study, our study also confirmed that FNDC5/irisin could be trafficked to osteoblasts via exosomes. Furthermore, we verified that FNDC5/irisin‐enriched exosomes entered osteoblasts through caveolae‐mediated endocytosis dependent on Cav1. Cav1 is a membrane protein located at various membrane structures, such as the cell membrane and Golgi apparatus membrane, and forms the core structural component of caveolae (Parton, 2018). Caveolae, which are known to function as endocytic carriers have been implicated in membrane trafficking via endocytosis, the process that allows cells to take up molecules from the environment (Hetmanski et al., 2019). Caveolae‐mediated endocytosis was shown to be one of the basic mechanisms of endocytosis that allows the internalization of exosomes (Sagar et al., 2016). CAV1 is an important cellular endocytic regulator during endocytosis. Scaffolding domains of Cav1 support the formation of caveolae and enable interaction with multiple proteins (Egger et al., 2020). Consistently, our study further supports that FNDC5/irisin binds to the scaffolding domain of Cav1 during endocytosis, which then binds to AMPKα and subsequently forms the FNDC5/irisin‐Cav1‐AMPKα complex to activate the AMPK pathway.

Our study demonstrated that proliferation and ferroptosis are the downstream processes of the irisin‐mediated protective effects on bone caused by activation of the AMPK pathway. Ferroptosis is an iron‐dependent nonapoptotic cell death characterized by iron overloading and increased lipid peroxide (Yoshida et al., 2019). Increased iron and lipid peroxide induces the Fenton reaction, leading to the production of highly reactive HO• radicals and cell death (Ashraf et al., 2020). A previous study indicated that irisin could suppress ferroptosis via activation of the Nrf2/GPX4 signalling axis (Wang et al., 2022). Importantly, our study further showed that Nrf2 is upregulated due to activation of the AMPK pathway, which also increases the transcription of Fpn. Fpn was identified in this study to be involved in irisin‐mediated antiferroptotic effects. Fpn is the only identified mammalian nonheme iron exporter responsible for the removal of iron from cells (Donovan et al., 2005). Increased Fpn due to irisin could ameliorate RSL3‐induced ferroptosis by promoting the removal of iron from osteoblasts. Concurrently, we also found that irisin could promote osteoblasts through regulation of the cell cycle. Consistent with our study, another report showed that irisin could increase macrophages in proliferating phases (Mazur‐Bialy, 2017). Our study supports that irisin serves as a regulator of the cell cycle and further demonstrates that HMOX1 is involved in regulating the cell cycle of osteoblasts by regulating the expression of CDK2/CycA2. Cyclin‐dependent kinases are holoenzymes responsible for the regulation of cellular processes that are composed of catalytic subunits and regulatory subunits (cyclin) (Harper & Adams, 2001). CDK2 could be activated by cyclin A (Cyc A), leading to the formation of a complex that regulates the progression of the S‐phase and performs essential functions in G2 and M‐phase cells (Chi et al., 2020). Increased CDK2/CycA2 could reverse the repression of pRb on E2F2, which is responsible for the transcription of genes involved in the cell cycle and DNA replication. Our study also verified that E2F2 is upregulated in osteoblasts after treatment with irisin in an HMOX1‐dependent manner, subsequently increasing the proliferation of osteoblasts via increases in the S and G2/M phases. Decreased ferroptosis and enhanced proliferation of osteoblasts together facilitate osteogenesis and subsequently rescue bone loss.

Our study reveals the reason for the increase in the release of FNDC5/irisin during exercise. Our results identified two polyunsaturated fatty acids, AA and DHA, as regulators that upregulate FNDC5/irisin in response to exercise. Consistent with our study, a previous study showed that rats fed high corn oil diets exhibited high levels of PGC1α and AA, while PUFAs can upregulate the expression of PGC1α in skeletal muscle cells in vitro (Rodriguez‐Cruz et al., 2006; Staiger et al., 2005). PGC1α is a transcriptional coactivator that has been demonstrated to interact with transcription factors to activate the transcription of FNDC5. In this study, nontargeted metabolomic profiling was applied to investigate alterations in metabolomic signatures in skeletal muscles in Ovx mice with or without treadmill exercise, indicating that AA and DHA were upregulated in exercised mice. Further studies showed that AA and DHA could increase the cell viability of myotubes and promote the expression and release of irisin. Previous studies have also shown that dietary supplementation of AA and DHA could suppress osteoporosis and reduce hip fracture risk in elderly individuals, thereby highlighting the potential bone‐protective properties of AA and DHA (Farina et al., 2011).

In conclusion, exercise increases the secretion of irisin in skeletal muscle through the upregulation of AA and DHA. FNDC5/irisin is then trafficked to osteoblasts via exosomes and enters osteoblasts through caveolae‐mediated endocytosis. In osteoblasts, irisin interacts with Cav1 and AMPKα to activate the AMPK pathway, which subsequently promotes proliferation and suppresses ferroptosis of osteoblasts dependent on Fpn and Hmox1. These findings provide novel insights into the effects of exercise on bone health and offer a new perspective for the treatment of osteoporosis based on irisin‐enriched exosomes.

## AUTHOR CONTRIBUTIONS

Lin Tao, Jinpeng Wang, and Ke Wang: conceptualization, methodology, validation, formal analysis, resources, writing—original draft and visualization. Qichang Liu: methodology, validation, resources and data curation. Hongyang Li: validation and validation. Chunjian Gu: data curation. Site Xu: validation and resources. Yue Zhu: conceptualization, writing—review and editing, supervision, project administration and funding acquisition.

## FUNDING INFORMATION

This work was supported by the National Natural Sciences Foundation of China (82072387 and 32200943).

## CONFLICT OF INTEREST STATEMENT

The authors are not aware of any affiliations, memberships, funding or financial holdings that might be perceived as affecting the objectivity of this study.

## Supporting information


Figure S1.

Figure S2.

Figure S3.

Table S1.

## Data Availability

Not available.
