# Supplementary figures and images for: Exerkine FNDC5/irisin‐enriched exosomes promote proliferation and inhibit ferroptosis of osteoblasts through interaction with Caveolin‐1
Source: Aging Cell. 2024 Apr 30;23(8):e14181. doi: 10.1111/acel.14181 (PMC11320359; doi:10.1111/acel.14181)

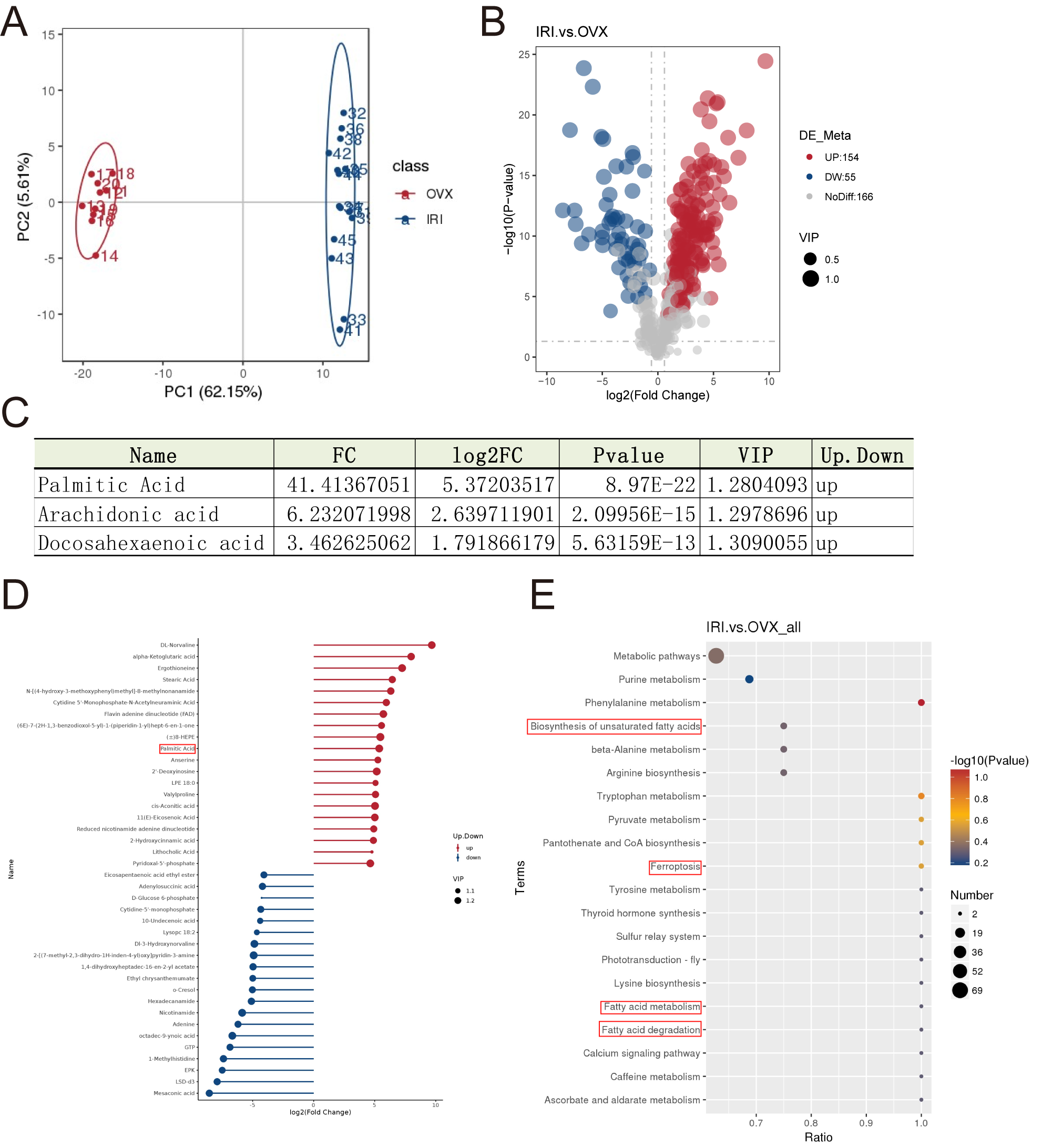

Supplement: Supplementary file 1 — Figure S1. [file ACEL-23-e14181-s004.tif]

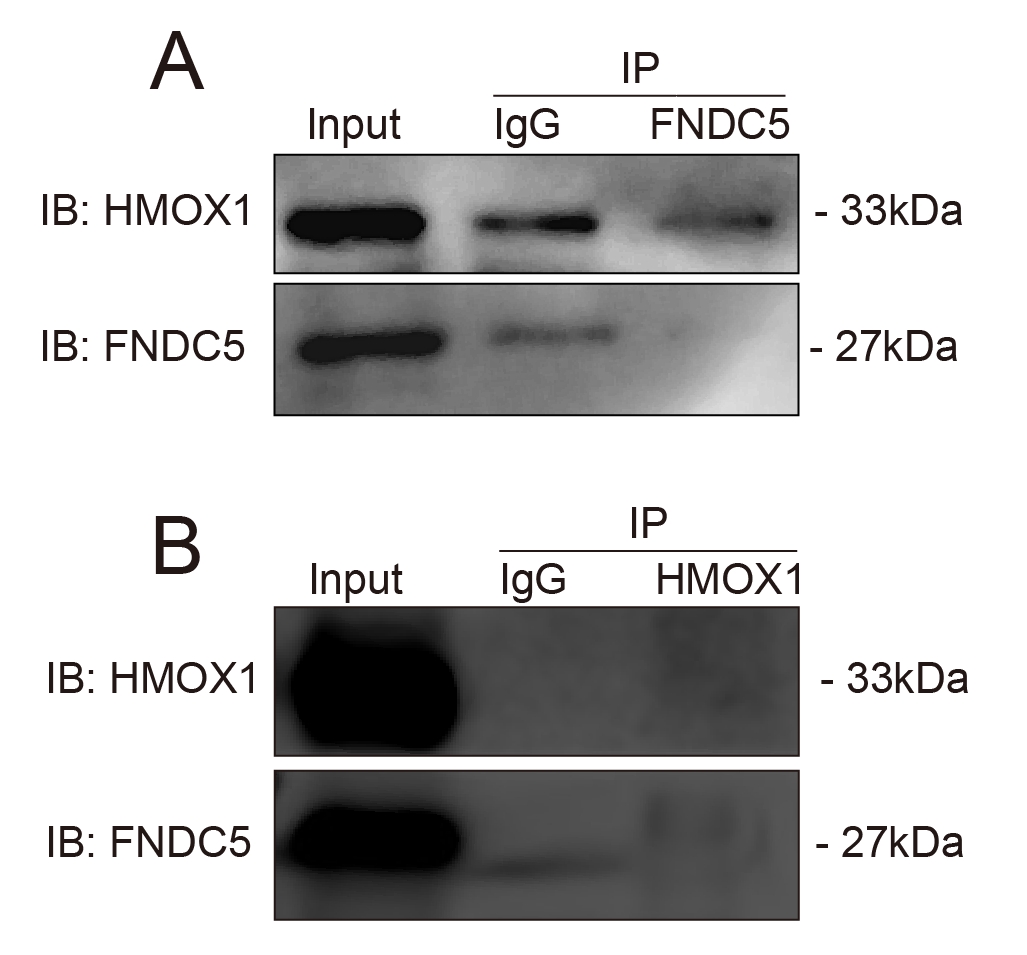

Supplement: Supplementary file 2 — Figure S2. [file ACEL-23-e14181-s001.tif]

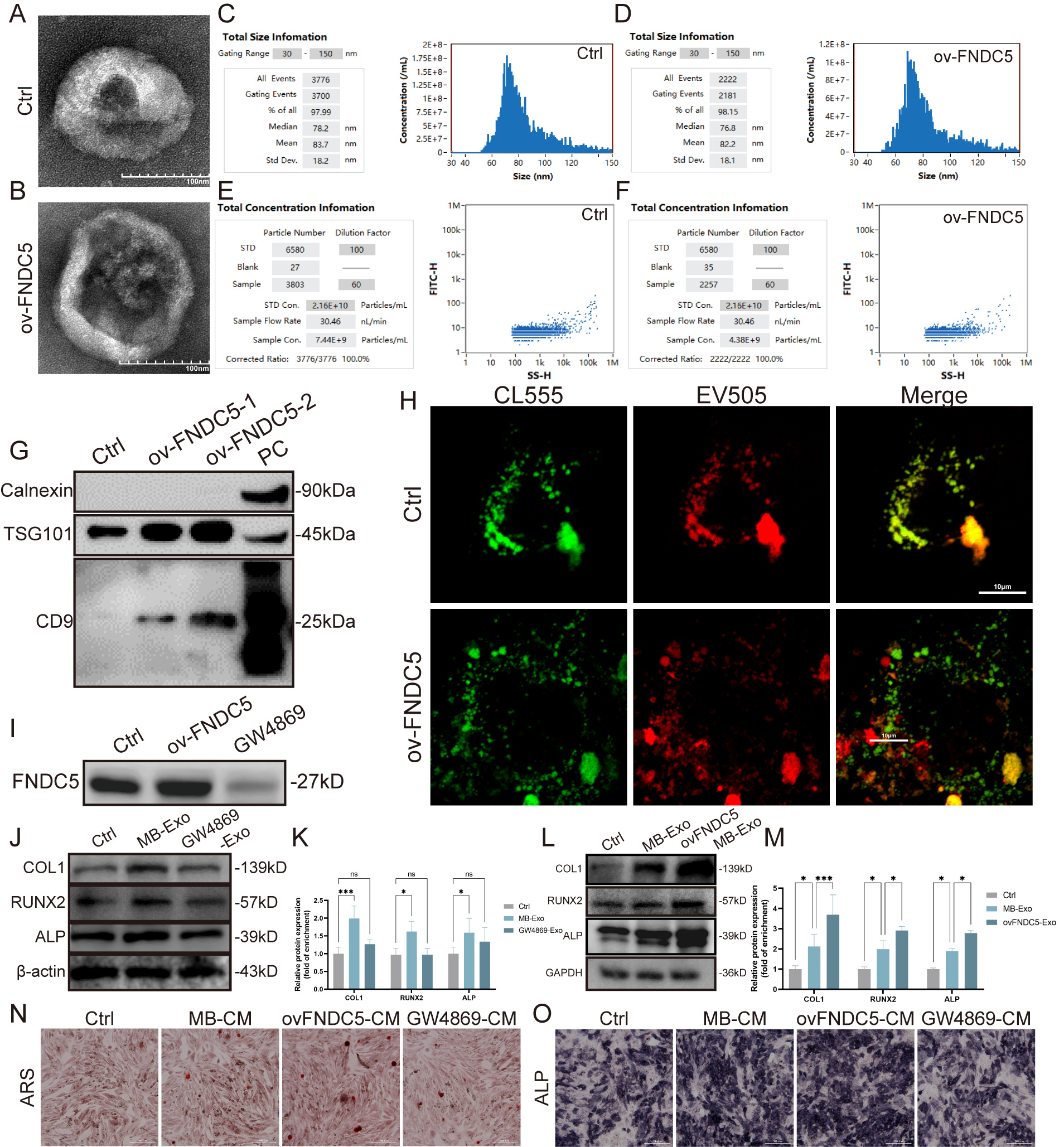

Supplement: Supplementary file 3 — Figure S3. [file ACEL-23-e14181-s003.tif]
