# Supplementary material for: Exerkine FNDC5/irisin‐enriched exosomes promote proliferation and inhibit ferroptosis of osteoblasts through interaction with Caveolin‐1
Source: Aging Cell. 2024 Apr 30;23(8):e14181. doi: 10.1111/acel.14181 (PMC11320359; doi:10.1111/acel.14181)
Supplement: Supplementary file 4 — Table S1. [file ACEL-23-e14181-s002.docx]

Table S1. Sequences of siRNAs and plasmids employed in this study.

| **siRNA/plasmid** | **sequence (5'-3')** |
| --- | --- |
| FNDC5-siRNA #1 | GGUUCAUUCAGGAGGUGAACATT |
| FNDC5-siRNA #2 | GUGAGCCUGUGCUCUUCAAGATT |
| FNDC5-siRNA #3 | AGGACAACGAGCCCAAUAACATT |
| Cav1-siRNA | CGACGUGGUCAAGAUUGACUUTT |
| HMOX1-siRNA #1 | GAGAAUGCUGAGUUCAUGAGGTT |
| HMOX1-siRNA #2 | CAGAAGAGCUGCACCGCAAGGTT |
| HMOX1-siRNA #3 | CCAAGUUCAAGCAGCUCUACCTT |
| Fpn-siRNA #1 | CUAAAGAUACUGAGCCAAATT |
| Fpn-siRNA #2 | UGGUGGAACUCUAUGGAAATT |
| Fpn-siRNA #3 | AGAAUGAGCUCCUGACCAUTT |
| FNDC5-overexpression plasmid | ATGCCCCCAGGGCCGTGCGCCTGGCCGCCCCGCGCCGCGCTCCGCCTGTGGCTAGGCTGCGTCTGCTTCGCGCTGGTGCAGGCGGACAGCCCCTCAGCCCCTGTGAACGTGACCGTCCGGCACCTCAAGGCCAACTCTGCCGTGGTCAGCTGGGATGTCCTGGAGGATGAAGTGGTCATTGGCTTTGCCATCTCTCAGCAGAAGAAGGATGTGCGGATGCTCCGGTTCATTCAGGAGGTGAACACCACCACCCGGTCCTGCGCTCTCTGGGACCTGGAGGAGGACACAGAATATATCGTCCATGTGCAGGCCATCTCCATCCAGGGACAGAGCCCAGCCAGTGAGCCTGTGCTCTTCAAGACCCCACGCGAGGCTGAAAAGATGGCCTCAAAGAACAAAGATGAGGTGACCATGAAGGAGATGGGGAGGAACCAGCAGCTGCGAACGGGGGAGGTGCTGATCATTGTTGTGGTCCTCTTCATGTGGGCAGGTGTTATAGCTCTCTTCTGCCGCCAGTATGATATCATCAAGGACAACGAGCCCAATAACAACAAGGAGAAAACCAAGAGCGCATCAGAAACCAGCACACCGGAGCATCAGGGTGGGGGTCTCCTCCGCAGCAAGATA |
| Cav1-overexpression plasmid | ATGTCTGGGGGCAAATACGTAGACTCCGAGGGACATCTCTACACTGTTCCCATCCGGGAACAGGGCAACATCTACAAGCCCAACAACAAGGCCATGGCAGACGAGGTGACTGAGAAGCAAGTGTATGACGCGCACACCAAGGAGATTGACCTGGTCAACCGCGACCCCAAGCATCTCAACGACGACGTGGTCAAGATTGACTTTGAAGATGTGATTGCAGAACCAGAAGGGACACACAGTTTCGACGGCATCTGGAAGGCCAGCTTCACCACCTTCACTGTGACAAAATATTGGTTTTACCGCTTGTTGTCTACGATCTTCGGCATCCCAATGGCACTCATCTGGGGCATTTACTTTGCCATTCTCTCCTTCCTGCACATCTGGGCGGTTGTACCGTGCATCAAGAGCTTCCTGATTGAGATTCAGTGCATCAGCCGCGTCTACTCCATCTACGTCCATACCTTCTGCGATCCACTCTTTGAAGCTATTGGCAAGATATTCAGCAACATCCGCATCAGCACGCAGAAAGAGATA |
